# Re–Os geochronology for sulfides and organic-rich sediments

**DOI:** 10.1093/nsr/nwaf300

**Published:** 2025-07-23

**Authors:** Yang Li, Stijn Glorie, David Selby

**Affiliations:** SKLab-DeepMine and MOEKLab-OBCE, School of Earth and Space Sciences, Peking University, Beijing 100871, China; Xinjiang Institute of Ecology and Geography, Chinese Academy of Sciences, Urumqi 830011, China; London Geochronology Centre, Department of Earth Sciences, University College London, London WC1E 6BT, UK; Department of Earth Sciences, the University of Adelaide, Adelaide 5005, Australia; Department of Earth Sciences, Durham University, Durham DH1 3LE, UK

**Keywords:** molybdenite, pyrite, shale, isochron, high-precision dating, imaging-guided

## Abstract

Rhenium and osmium are both siderophilic and chalcophilic, exhibiting a strong affinity for organic-rich materials. This makes the Re–Os chronometer a valuable complement to geochronometers based on lithophile elements. In this review, we begin by discussing how the elemental abundances and isotopic compositions impact sample selection, analytical strategy, and data interpretation. We then provide an overview of how ^187^Os/^188^Os ratios can be used to trace geological processes, followed by a summary of the analytical protocols commonly used in Re–Os geochemistry. We also examine key challenges in isochron dating, including the identification and avoidance of pitfalls such as mixing lines, and inherited initial slopes. We further demonstrate that petrographic and geochemical studies can be very helpful for accurately dating sulfides with contrasting initial ^187^Os/^188^Os values and/or ages. With state-of-the-art Re–Os dating technique reaching precisions up to 0.05% for molybdenites and 1% for organic-rich sedimentary rocks, it is now possible to resolve the rapid and episodic nature of ore formation, and to investigate the dynamics of environment–life coevolution with unprecedented detail. We conclude this review by outlining future directions for Re–Os geochronology, including developing imaging-guided Re–Os dating techniques for organic-rich sediments, sharpening the *in situ* Re–Os dating method, and fully integrating the Re–Os geochronometer into the EarthTime initiative.

## Teaser

The Re–Os isotope system holds Earth's memory in deep-time—from the heartbeats of ore formation to the pathways of hydrocarbons, from climate change to life evolution, from hot magmas to shining meteorites. This review explores how Re–Os isotopes trace these processes across Earth's mantle, crust and oceans.

## INTRODUCTION

Rhenium (Re) and Osmium (Os) are siderophile (iron-loving) and chalcophile (sulfur-loving) elements that additionally exhibit a strong affinity for organic-rich materials. As such, Re and Os are commonly enriched in sulfide minerals, organic-rich sedimentary rocks, hydrocarbons (e.g. crude oil, bitumen), coal and graphite [1–10]. These characteristics distinguish the Re–Os system from more commonly used radiogenic chronometers, such as Rb–Sr, Lu–Hf, Sm–Nd and U–Th–Pb, which primarily involve lithophile elements hosted in silicate minerals.

Although Os was discovered in 1803 [11], Re was not identified until over a century later, in 1908 [12]. Consistent determination of the ^187^Re half-life was only achieved in the 1990s [13]. As one of the most recently developed radiogenic chronometers, the Re–Os system has proved to be a valuable addition to the geochronological toolbox, offering a complementary perspective to traditional methods based primarily on lithophile elements.

**Figure 1.**
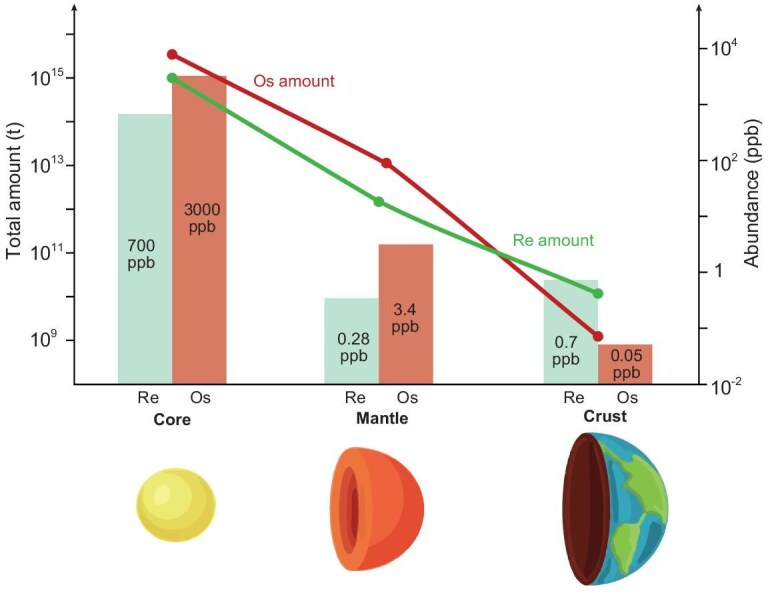
Distribution of Re and Os in Earth's core, mantle and crust. Nearly all Re and Os (99.86%–99.94%) are sequestered into Earth's core, leaving a Re and Os depleted bulk silicate Earth. During mantle partial melting, incompatible Re preferentially enters into the melt, resulting in a Re-enriched and Os-depleted continental crust (∼0.7 ppb Re and ∼0.05 ppb Os) and an Os-enriched and Re-depleted mantle (∼0.28 ppb Re and ∼3.4 ppb Os). Note that the Re and Os abundances are in ppm levels for the core, but in ppb levels for the mantle and crust. See Table 1 for data.

The siderophilic nature of Re and Os leads to their preferential partitioning into Earth's core during planetary differentiation (Fig. 1). Approximately 99.86% of Re and 99.94% of Os are sequestered into the core (Table 1), resulting in significant depletion of both elements in the bulk silicate Earth (mantle and crust) [14–17]. The distribution of Re and Os between the mantle and crust is primarily controlled by their contrasting geochemical behaviors during partial melting [18,19]. Rhenium behaves incompatibly, preferentially entering the melt phase, whereas Os is compatible and largely remains in the residual crystalline phases [19]. Over time, this leads to significantly higher Re but lower Os concentrations in the continental crust (∼0.7 ppb Re and ∼0.05 ppb Os; Fig. 1, Table 1) compared to the depleted mantle (∼0.28 ppb Re and ∼3.4 ppb Os). Due to the elevated Re/Os ratio of the crust relative to the mantle (∼14 vs ∼0.08, Fig. 1), the β-decay of ^187^Re to ^187^Os leads to progressively more radiogenic ^187^Os/^188^Os ratios in the continental crust over time, making ^187^Os/^188^Os a powerful tracer for geological processes.

**Table 1.** Re and Os abundances in the Earth's core, mantle and crust, and the isotopic composition of commonly dated materials.

	Re abundance	Os abundance	Mass (tonnes)	Re budget (tonnes)	Os budget (tonnes)	^187^Re/^188^Os
Core	∼0.70 ppm [16,17]	∼3.0 ppm [16,17]	1.9 × 10^21^ [16]	1.4 × 10^15^	5.8 × 10^15^	
Mantle	∼0.28 ppb [17]	∼3.4 ppb [17]	4.0 × 10^21^ [16]	1.1 × 10^12^	1.4 × 10^13^	
Crust	0.40–1.0 ppb [14]	5.0 × 10^−2^ ppb [15]	3.0 × 10^19^ [16]	2.1 × 10^10^	1.5 × 10^9^	
Molybdenite	4.2 × 10^−4^ –1.0 × 10^4^ ppm					∞
Pyrite	1.2 × 10^−3^ –4.6 × 10^4^ ppb					3.5 × 10^−1^–2.7 × 10^6^
ORS	3.0 × 10^−1^–1.4 × 10^3^ ppb					1.1 × 10^0^–6.3 × 10^3^
Crude oil	1.0 × 10^−2^–1.5 × 10^3^ ppb					1.5 × 10^0^–1.1 × 10^4^
Bitumen	3.0 × 10^−2^–4.4 × 10^2^ ppb					2.5 × 10^1^–1.8 × 10^3^

Data sources for molybdenite, pyrite, organic-rich sediments (ORS), crude oil and bitumen are provided in Table S1.

The Re–Os chronometer has been successfully applied to a wide range of materials from both the Earth and the solar system, including meteorites [13], sulfide minerals [6,8,9,20–26], organic-rich sedimentary rocks [27–30] and even hydrocarbons [31,32]. These applications have produced a wide range in Re–Os ages, spanning from as old as 4.56 Ga for meteorites [13] to as young as ∼78 years for rheniite [33]. In addition to enhancing our understanding on planetary formation [13], mantle–crust interactions [34,35] and the environment-life coevolution [29,36,37], the Re–Os chronometer has also proved to be essential in providing absolute time constraints for, and identifying the source rocks of metallic ore deposits and hydrocarbons [25,26,38–40].

This review begins with a concise summary of the fundamentals of Re–Os geochemistry and geochronology, then highlights key advances over the past two decades, and concludes with a discussion on potential directions for future research. For more comprehensive accounts of the historical development, analytical challenges and broad applications of the Re–Os chronometer, readers are referred to previous reviews [e.g. 41,42–44].

### RE–OS SYSTEMATICS

#### Re and Os elemental and isotopic composition

**Figure 2.**
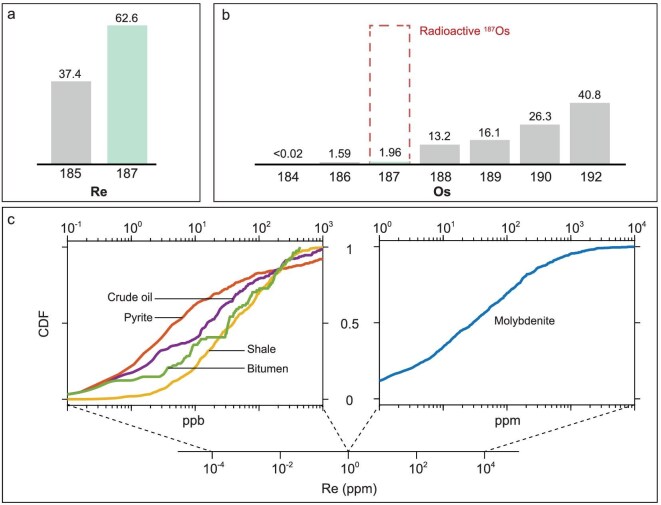
Elemental and isotopic composition of Re and Os. (a, b) Isotopic composition of Re and Os in natural samples. The abundance of radiogenic ^187^Os varies across samples, with higher values generally found in those with greater Re concentrations and older geological ages. (c) Rhenium concentrations in molybdenite, pyrite, shale, bitumen and crude oil. Molybdenite displays the greatest Re concentrations, ranging from sub-ppm to % levels. In contrast, Re contents in pyrite, organic-rich sediments (e.g. shale), bitumen and crude oil are typically three orders of magnitude lower, ranging from hundreds of ppt to ppm levels. CDF, cumulative distribution function. Data sources and references are provided in the Supplementary data (Table S1; the Re–Os systematics for molybdenite, pyrite, organic-rich sediments, bitumen and crude oil compiled from the literature. The data are presented in Figs 2 and 3. References for the data are also given in Table S1.)

Rhenium has two naturally occurring isotopes: ^185^Re and ^187^Re, with an isotopic abundance of 37.4% and 62.6%, respectively (Fig. 2a). Of these, ^187^Re is radiogenic and decays to ^187^Os [45]. The general formula for Re–Os geochronology is given in Equation (1):


(1) \begin{eqnarray*}
{}^{187}{\mathrm{O}}{{\mathrm{s}}}_{{measured}} = {\ }^{187}{\mathrm{O}}{{\mathrm{s}}}_{{initial}}\
&+& {\ }^{187}{\mathrm{R}}{{\mathrm{e}}}_{{measured}} \\
\times \ \left( {{e}^{{\mathrm{\lambda t}}} - \ 1} \right).
\end{eqnarray*}

In Equation (1), *t* represents the age of the sample, and λ is the decay constant of ^187^Re. The terms ^187^Re_measured_ and ^187^Os_measured_ refer to the measured isotopic abundances, while ^187^Os_initial_ denotes the molar abundance of non-radiogenic ^187^Os, namely the Os incorporated into the mineral at the time of its formation, commonly referred to as ‘common Os’. Using a decay constant (λ) of (1.666 ± 0.017) × 10^−11^ year^−1^ [13], the half-life of ^187^Re is approximately 41.6 billion years—around nine times the age of the Earth.

The reliable application of any radiogenic chronometer depends on a thorough understanding of the elemental and isotopic composition of the targeted samples (Fig. 2). Given the extremely low concentrations of Re in both the mantle and crust, coupled with the slow decay rate of ^187^Re, geochronological studies must focus on minerals or phases that are significantly enriched in Re. To date, the most extensively investigated materials include molybdenite, pyrite, organic-rich sedimentary rocks, bitumen and crude oil [41,42].

Rhenium rarely forms discrete minerals in nature, with the notable exception of rheniite (ReS_2_) [33]. Instead, Re is typically concentrated in molybdenite (MoS_2_), where its abundance ranges from sub-ppm to % levels (Fig. 2c). The high Re concentrations in molybdenite are generally attributed to the substitution of Re for Mo in the crystal lattice, owing to (i) the similar ionic radii of Re^4+^ and Mo^4+^ (0.63 and 0.65 Å, respectively) and (ii) the strong partitioning of Re into molybdenite relative to coexisting sulfides (e.g. pyrite, chalcopyrite) and/or silicate minerals. Combined with the typically negligible concentrations of common Os in molybdenites, i.e. ^187^Os_initial_ is generally negligible, molybdenite Re–Os geochronology is widely applied as a high-precision, single-mineral chronometer with high accuracy [46–49].

Another commonly studied mineral in Re-Os geochronology is pyrite [4,22,26,38,50], although other sulfide minerals such as arsenopyrite [23,51,52], bornite [25,53,54], chalcopyrite [55–57], cobaltite [6] and safflorite [24] have also been used. Pyrites typically contain low Re concentrations (e.g. [58]), ranging from a few ppb up to ppm levels (Fig. 2c), while its common Os content is highly variable, from negligible amounts to tens of ppb.

Organic-rich materials, such as organic-rich sedimentary rocks, bitumen and crude oil, contain the lowest Re concentrations among commonly dated samples (Fig. 2c), and typically incorporate initial Os at sub-ppb to ppb levels. Though not exclusively, the presence of elevated Mo concentrations and high total organic carbon in organic-rich sediments may indicate elevated Re abundances [59].

**Figure 3.**
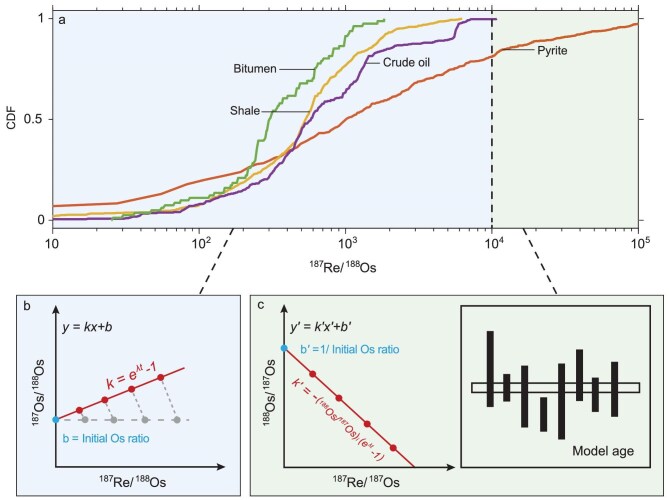
Sample ^187^Re/^188^Os ratios govern data visualization and interpretation. (a) The ^187^Re/^188^Os ratios in pyrite, organic-rich sediments (e.g. shale), bitumen and crude oil typically range from ∼10 to 10^4^. Molybdenite contains high Re and exceptionally low common Os, resulting in extremely high ^187^Re/^188^Os ratios; thus, they are not shown. Some pyrites also exhibit ^187^Re/^188^Os ratios much higher than 10^4^ and are referred to as low-level (Re), highly radiogenic (^187^Os) samples (LLHR [4]). Here, LLHR samples are defined as those with ^187^Re/^188^Os > 10^4^. (b) For samples with non-negligible common Os (e.g. ^187^Re/^188^Os < 10^4^), such as organic-rich sediments, bitumen and crude oil, several co-genetic samples must be analyzed to obtain an isochron in the ^187^Re/^188^Os vs. ^187^Os/^188^Os space, as described by Equation (4). (c) For samples with negligible common Os (e.g. ^187^Re/^188^Os > 10^4^), such as molybdenite and certain pyrites, a model age can be calculated for each sample using Equation (3); Then a weighted average can be calculated for multiple co-genetic samples. Given the imprecise measurement of ^188^Os at very low concentrations, the traditional isochron approach is not recommended unless uncertainties are properly propagated with the consideration of the error correlation (ρ) [160,161]. Alternatively, an inverse isochron is recommended by plotting ^187^Re/^187^Os vs. ^188^Os/^187^Os [63]. Relevant data and references are provided in Table S1.

Because of the large variations in Re and Os elemental and isotopic concentrations among these samples (Figs 2 and 3, Table 1), strategies in Re-Os geochronology must match specific sample types. These strategies encompass sample collection, processing, isotopic analysis, data reduction and interpretation—from samples to data, to dates, and ultimately to ages. For example, samples with high Re concentrations require smaller sample sizes to achieve a given precision in isotopic measurements compared to those with lower Re content and/or younger ages. In addition to Re abundance, isotopic composition is also a critical factor, although it is typically unknown prior to analysis. To assist in methodological planning, here we have compiled literature data (Fig. 3a) to provide a framework for guiding Re–Os geochronological studies.

The mathematical expression for the age (*t*) can be derived by rearranging Equation (1):


(2) \begin{eqnarray*}
t = \frac{{{\rm ln}\left( {\left[ {\frac{{{}^{187}{\rm Os}_{\rm {measured}} - {}^{187}{\rm Os}_{\rm {initial}}}}{{{}^{187}{\rm Re}_{\rm {measured}}}}} \right] + 1} \right)}}{\lambda }.
\end{eqnarray*}

Equation (2) contains two unknowns (*t* and ^187^Os_initial_), which can be solved using either a single sample or multiple samples, depending on the abundances of ^187^Os_initial_.

#### Samples with high ^187^Re/^188^Os ratios (>10^4^)

If ^187^Os_initial_ is negligible, *t* can be determined from a single sample using Equation (3):


(3) \begin{eqnarray*}
t = \frac{{{\rm ln}\left( {\left[ {\frac{{{}_{}^{187}{\rm Os}_{\rm {measured}}}}{{{}_{}^{187}{\rm Re}_{\rm {measured}}}}} \right] + 1} \right)}}{\lambda }.
\end{eqnarray*}

Most molybdenites and some pyrites, which have high Re abundances but extremely low common Os content, meet this criterion [4,53], as indicated by their very high ^187^Re/^188^Os ratios (>10^4^, Fig. 3a).

Dates calculated from Equation (3) are referred to as model ages, based on the assumption that ^187^Os_initial_ is negligible. For samples with ^187^Re/^188^Os ratios of >10^4^, this assumption introduces an overestimation of <0.09 and <0.05 Ma for samples aged at 100 Ma and 4000 Ma, respectively, which are negligible for an analytical precision of 0.1%. However, this may become a concern for younger samples when high accuracy is required. For example, for samples with ^187^Re/^188^Os ratios of 10^4^ but ages of 1 Ma and 10 Ma, the model ages will be overestimated by approximately 10% and 1%, respectively.

The assumption that common Os is negligible can be tested analytically using a double spike [60,61]. Alternatively, multiple samples with an identical age (e.g. molybdenites from the same vein) may be analyzed as an independent examination [49]. If samples with lower Re content systematically yield older ages, this provides strong evidence that the assumption of negligible common Os is not satisfied.

When the assumption of negligible common Os is valid, multiple co-genetic samples can be used to calculate a weighted mean age with improved precision (Fig. 3c). Alternatively, an isochron age can be derived from a ^187^Re vs ^187^Os diagram, which is effectively equivalent to the weighted mean of model ages (Fig. 3c).

Since isotope ratios are measured with a much higher precision and accuracy then isotope abundances, in practice, Equation (1) is divided by a stable isotope of Os, and ^188^Os is the typical choice, with a natural isotopic abundance of 13.2%.


(4) \begin{eqnarray*}
{\left[ {\frac{{{}^{187}{\rm Os}}}{{{}^{188}{\rm Os}}}} \right]}_{\rm {measured}} = {\left[ {\frac{{{}^{187}{\rm Os}}}{{{}^{188}{\rm Os}}}} \right]}_{\rm {initial}}\\
+ {\left[ {\frac{{{}^{187}{\rm Re}}}{{{}^{188}{\rm Os}}}} \right]}_{\rm {measured}} \times \left( {{e}^{\lambda t} - 1} \right)
\end{eqnarray*}

In Equation (4), the uncertainties of the ^187^Re/^188^Os and ^187^Os/^188^Os ratios are correlated, as both share a common denominator arising from the ^188^Os measurement. However, this approach is not recommended for samples with very high ^187^Re/^188^Os ratios unless uncertainty propagation is accurately performed with explicit consideration of error correlation [62].

Specifically, because ^188^Os concentrations are typically several orders of magnitude lower than those of ^187^Re and ^187^Os, the uncertainties in the ^187^Re/^188^Os and ^187^Os/^188^Os ratios are dominated by the imprecise measurements of ^188^Os [4], and are therefore highly correlated (e.g. >0.99). For datasets with very high ^187^Re/^188^Os ratios, plotting an isochron without accounting for error correlation (i.e. assuming a correlation coefficient of zero) should be avoided, as this will bias the isochron age and lead to overestimated uncertainties. Given the extremely low concentrations of common Os, the isochron's *y*-intercept is expected to have large uncertainties [53], and using it as a tracer is not recommended.

An improved method for datasets with very high ^187^Re/^188^Os ratios—and thus highly correlated uncertainties between the ^187^Re/^188^Os and ^187^Os/^188^Os ratios—is the inverse isochron approach (Fig. 3c), which involves plotting the data in ^187^Re/^187^Os vs ^188^Os/^187^Os space [63]. This method is also recommended for isochron studies with moderate to low levels of correlated uncertainties as it provides a superior visualization of dispersion.

#### Samples with moderate to low ^187^Re/^188^Os ratios (<10^4^)

For samples with non-negligible common Os—such as most pyrite, organic-rich sediments, bitumen and crude oil (Fig. 3a)—a model age cannot be obtained using Equation (3). Instead, several co-genetic samples must be analyzed using the isochron approach (Fig. 3b), following Equation (4).

In addition to yielding an age from its slope (e^λt^ − 1), the isochron approach also provides the isotopic composition for common Os (Os_i_ = ^187^Os/^188^Os_initial_) from its *y*-intercept (Fig. 3b). Although the inclusion of common Os introduces an additional dimensional of information, it comes at a cost—limiting the precision of age determinations. This explains why isochron ages are typically less precise than the model ages described above.

## OS ISOTOPE AS A TRACER FOR GEOLOGICAL PROCESSES

**Figure 4.**
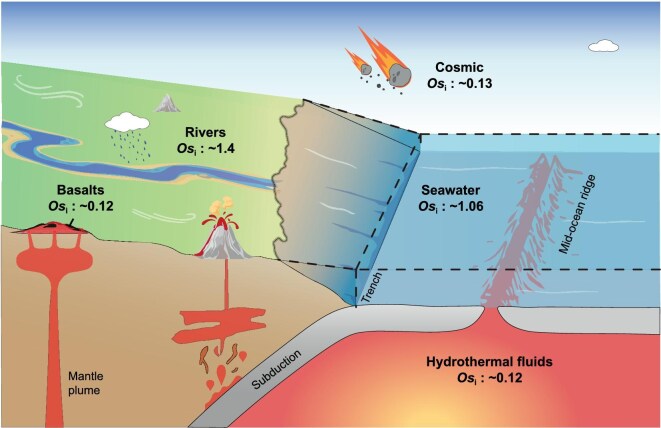
Osmium isotope composition (Os_i_, i.e. ^187^Os/^188^Os_initial_) of common geological reservoirs. Basalts derived from the present-day mantle have an average Os_i_ of ∼0.12, representing the lower limit among geological reservoirs. The modern ocean has an Os_i_ of ∼1.06, reflecting a balance between more radiogenic continental river runoff (average Os_i_ *∼* 1.4) and hydrothermal inputs at middle-ocean ridges (average Os_i_ *∼* 0.12). Cosmic dust and meteorites typically exhibit an Os_i_ of ∼0.13.

The preferential enrichment of Re in the continental crust underpins the use of ^187^Os/^188^Os ratios as tracers of crustal processes (Fig. 4). At present, with an average radiogenic Os_i_ of approximately 1.4, the continental crust is distinctly different from mantle-derived materials—such as basalts and hydrothermal fluids at middle-ocean ridges—which typically exhibit an Os_i_ of 0.12. In this regard, The Re–Os isotope system has been shown to be a powerful tool for tracking magma evolution during basaltic eruptions, owing to its exceptional sensitivity to crustal assimilation. Specifically, it can detect the incorporation of crustal materials with high Re/Os ratios and radiogenic ^187^Os/^188^Os values that often go unnoticed by conventional isotope systems such as Sr–Nd–Pb. For example, Os_i_ values of 0.188 and 0.132 were used to trace the degree of crustal contamination in the 2021 Fagradalsfjall basaltic eruption in Iceland [64].

The residence time of Os in the ocean is relatively short, with estimates ranging from 3–4 to 35–50 kyr [65–68], making Os isotopes a sensitive proxy for investigating a range of geological processes, though a short residence time may hinder its application in tracing some global events. An increased flux of mantle-derived materials to the global oceans—either through enhanced weathering of flood basalts or increased hydrothermal input at middle-ocean ridges and large igneous provinces—can be traced using Os_i_ signatures preserved in organic-rich sediments (Fig. 4), such as during oceanic anoxic events [69–71].

The rate of continental weathering also can be tracked using the Os_i_ record [72]. For example, postglacial strata younger than 662 Ma exhibiting radiogenic Os_i_ (∼0.54) have been used to infer intense silicate weathering during the post–Snowball Earth hothouse interval [30]. Radiogenic Os_i_ values from the Lantian and other Ediacaran shales (>1.0) have been attributed to oxidative weathering of the upper continental crust during the early to middle Ediacaran [36], potentially serving as a trigger for biological evolution and oceanic atmosphere oxygenation [73].

Cosmic inputs exhibit chondrite-like Os_i_ values (∼0.13) and can be used to indicate the presence of impact events [74,75]. As discussed above, mantle-derived hydrothermal fluid, basalt weathering and cosmic input all contribute to lowering oceanic Os_i_ (Fig. 4). Therefore, additional geological and geochemical evidence is essential to reliably distinguish these potential sources of Os_i_ fluctuations [45]. For meteorites, although their Os isotope signatures are often indistinguishable from those of the Earth's mantle, they are highly enriched in platinum-group elements (PGEs)—by factors of up to 1000 relative to mantle rocks and 100 000 compared to average crustal compositions [76]. This unique combination means that even trace amounts of meteoritic material can generate a measurably shift in marine ^187^Os/^188^Os ratios and PGE concentration. A classic example is the K–Pg boundary, where both a pronounced Ir spike and a drop in Os_i_ provide compelling evidence of an extraterrestrial impact. Such isotopic fingerprints have been used not only to estimate the magnitude and timing of meteoritic inputs to Earth's surface [77], but also to infer the type [78] and size [79] of the impacting bodies.

## ANALYTICAL PROCEDURES

**Figure 5.**
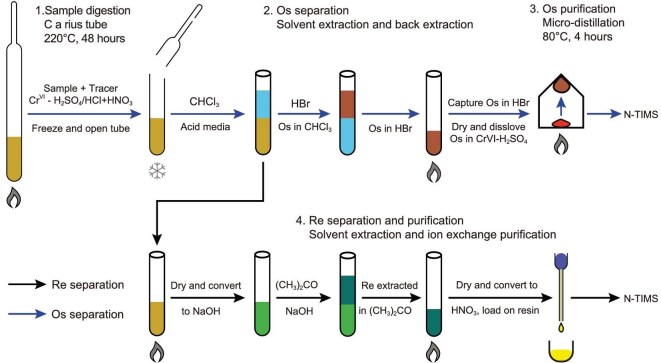
A representative analytical workflow for Re–Os geochronology. Re–Os geochronology begins with sample digestion using either HNO_3_–HCl (for sulfides, bitumen and crude oil) or CrO_3_–H_2_SO_4_ (for organic-rich sediments) as the digestion medium. The Carius-tube technique is preferred to minimize Os loss during digestion, and an oxidizing environment is essential to ensure complete isotopic equilibration between spike and sample Os. Osmium is separated from the digestion medium by solvent extraction using CHCl_3_, followed by back-extraction with HBr and purification by micro-distillation, prior to isotopic analysis by NTIMS. Rhenium is converted to an NaOH medium and separated by solvent extraction using acetone [(CH_3_)_2_CO]. Further purification of Re is performed using ion exchange before isotopic analysis by NTIMS or MC-ICPMS. The tubes are for demonstration purposes only and are not actual containers used in the experiments.

The conventional analytical procedure (Fig. 5) for obtaining Re–Os dates is by isotope dilution. The new *in situ* approach is not addressed in this section but is discussed separately below. Sample digestion is a critical step in conventional Re–Os geochronology [80]. Isotope dilution requires complete isotope equilibrium between spike and sample, which is straightforward for Re but challenging for Os. This is because Os reacts with oxygen at ambient temperatures to form osmium tetroxide (OsO_4_), a volatile and highly toxic compound [81]. To ensure complete Os isotope equilibration between the spike and sample, digestion must occur under highly oxidizing conditions at elevated temperature (e.g., >200 °C) to convert all Os to its highest oxidization state (Os^8+^). This necessitates the use of an oxidizing medium, such as inverse aqua regia (2*HNO_3_–1*HCl), for sulfides, bitumen and crude oil, in combination with the Carius-tube technique [82]. Due to the unavoidable presence of detrital Re and Os, CrO_3_–H_2_SO_4_, rather than inverse aqua regia, is used for organic-rich sediments to preferentially release Re and Os from the hydrogenous component [1,28].

Solvent extraction (Fig. 5) is the standard method for isolating both elements—using CHCl_3_ to extract Os from the digestion medium and to extract Re from an NaOH solution [83–86]. A sparging technique may also be used to extract Os directly by introducing oxygen into Os-bearing solutions [87]. Osmium can be further purified via back-extraction and micro-distillation using HBr [84]. For Re, further purification is carried out using anion exchange chromatography [1,83].

The high ionization potential of Os (8.7 eV) and its low abundance (sub-ppb levels) hinder isotopic composition measurements using traditional thermal ionization mass spectrometry (TIMS) analysis as positive ions, thereby significantly limiting its application as a chronometer. Creaser *et al.* [88] and Völkening *et al.* [89] independently demonstrated that Re and Os can be ionized as negatively charged oxides (ReO_4_^–^ and OsO_3_^–^) using Ba(OH)_2_ and/or Ba(NO_3_)_2_ as an activator. This revolutionary approach led to a multi-fold increase in the ionization efficiency of Re and Os, greatly reducing the sample size required for Re–Os geochronology while improving both precision and accuracy. It represents a major milestone that catalyzed the widespread application of this chronometer over the past 30 years.

## CRITICAL CONSIDERATION FOR ACCURATE DATING

### Mixing line and the concentration test

**Figure 6.**
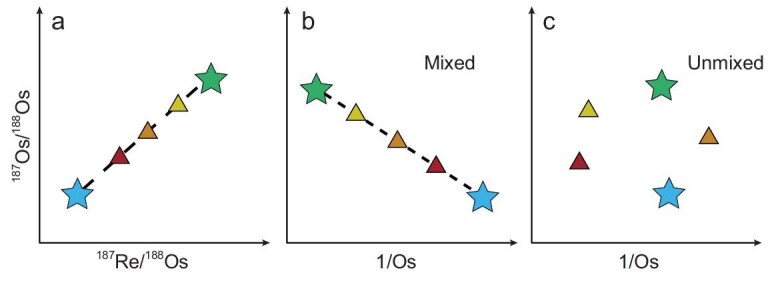
Distinguishing between isochron and mixing line. (a) An apparent ‘isochron’ can result from mixing two end-member components. (b, c) This can be identified using the 1/Os vs ^187^Os/^188^Os (also known as the 1/C test). A linear array in this plot indicates mixing rather than true age information. Note that the 1/C test is not applicable for LLHR samples.

A common pitfall in isochron dating is calculating ages from an apparent isochron produced by mixing two end-member components [90]. For example, water–rock interaction between hydrothermal fluids and country rocks can generate alteration assemblages with variable ^187^Re/^188^Os and ^187^Os/^188^Os ratios, which may define a linear trend (Fig. 6a). Additionally, analysis of different organic fractions of a single oil may also yield a linear trend.

Such linear trends maybe mixing lines without true chronological significance, which can be identified using the concentration test, i.e. by plotting 1/Os versus ^187^Os/^188^Os, where Os is defined by Equation (5):


(5) \begin{eqnarray*}
{\mathrm{Os }} &=& {}^{187}{\mathrm{O}}{{\mathrm{s}}}_{{\mathrm{radiogenic}}}\\
&& + \, \left[ {^{184, 186, 187, 188, 189, 190, 192}{\mathrm{O}}{{\mathrm{s}}}_{{\mathrm{initial}}}} \right].
\end{eqnarray*}

If a correlation is observed from the concentration test (Fig. 6b), it provides strong evidence that the apparent ‘isochron’ may in fact be a mixing line.

However, this test is not universally applicable. For samples with very low common Os content, where the total Os budget is dominated by radiogenic ^187^Os—such as molybdenites and pyrites with a ^187^Re/^188^Os ratio of >10^4^—a negative correlation between 1/Os and ^187^Os/^188^Os is expected. In such cases, the concentration test becomes invalid, as the observed trends reflect radiogenic ingrowth rather than mixing.

### Initial slope

When conducting isochron dating, it is essential that the samples share an identical formation age. However, in practice, samples with slightly different ages are sometimes used to achieve the necessary spread in the ^187^Re/^188^Os vs ^187^Os/^188^Os space for linear regression. For example, in Re–Os geochronology of organic-rich sedimentary rocks, sampling along a horizontal profile may yield a dataset with nearly uniform ^187^Re/^188^Os and ^187^Os/^188^Os ratios, making it unsuitable for isochron dating. To obtain the required variation, samples are typically collected along a vertical profile, in which case samples from the bottom layer were older and thus accumulated slightly more radiogenic ^187^Os prior to the deposition of upper layers. This results in an initial slope in the isochron diagram—either positive or negative depending on the relationship between Os_i_ and ^187^Re/^188^Os ratios—leading to either overestimated or underestimated dates. Similarly, variations in Os_i_ are another concern.

The impact of this initial slope can be significant for younger samples with a slow depositional rate [91,92] or those collected over an extended vertical interval but is generally negligible for older samples sampled over a short vertical range. In practice, the acceptable profile height should be evaluated on a case-by-case basis.

### Mixing multisource samples of the same age

**Figure 7.**
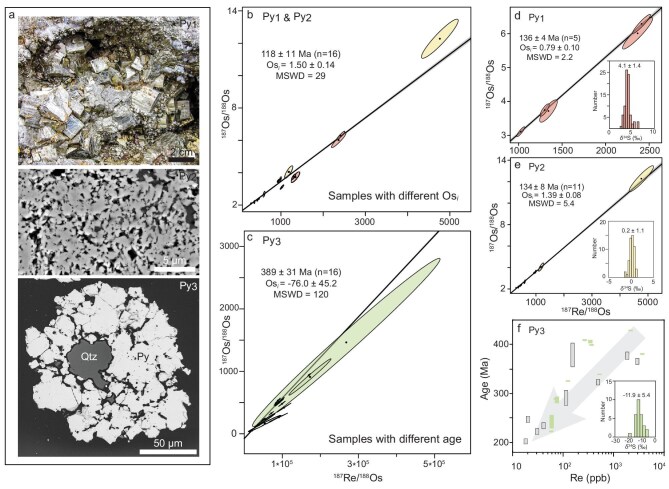
Dating pyrites with contrasting elemental and isotopic compositions. (a) Pyrites from the Xinqiao deposit include euhedral pyrites (py1, top panel) and colloform pyrites (py2, middle panel) within the stratabound massive orebody, as well as pyrites occurring in sandstone-hosted stockworks (py3, bottom panel). (b) Pyrites (py1 and py2) from the stratabound massive orebody define a 118 ± 11 Ma Re–Os errorchron (Os_i_ = 1.50 ± 0.14; MSWD = 29). (c) Pyrites (py3) from the sandstone-hosted stockworks yield a 389 ± 31 Re–Os errorchron (Os_i_ = −76 ± 45; MSWD = 120). Note that py3 pyrites are characterized as LLHR pyrite, displaying significantly higher ^187^Re/^188^Os ratios compared to py1 and py2 pyrites, and using the traditional isochron diagram is not recommended. (d) Euhedral pyrites (py1) yield a 136 ± 4 Ma Re–Os isochron (Os_i_ = 0.79 ± 0.10; MSWD = 2.2). (e) Colloform pyrites (py2) yield a 134 ± 8 Ma Re–Os isochron (Os_i_ = 1.39 ± 0.08; MSWD = 5.4). (f) Model ages from the stockwork pyrites (py3) vary from ∼380 Ma to 170 Ma and show a positive correlation with Re content, violating the prior of one population. Sulfur isotope data (inserts in d, e and f) demonstrate that these pyrites formed under different conditions. Py, pyrite; Qtz, quartz.

In addition to verifying that the samples used for isochron dating formed at the same time, another potential issue arises when mixing samples with different Os_i_ despite identical ages, which is a common problem when dating mineralization with extensive water–rock interaction [93]. The Re–Os dataset from the Xinqiao massive sulfide deposit in China [38] provides a compelling example for this scenario (Fig. 7).

This stratabound deposit features massive pyrite (py1) and colloform pyrite (py2) ores hosted in the Carboniferous limestone, and pyrite stockworks (py3) hosted in the Devonian sandstone. Although these pyrites (Fig. 7a) are genetically linked to the ∼138 Ma diorite [94], they exhibit different ^187^Re/^188^Os ratios and Os_i_ values (Fig. 7b and c). Py1 and py2 contain common Os with distinct Os_i_ values: ∼0.79 for py1 and ∼1.35 for py2 at 134–136 Ma (Fig. 7d and e). Py3 is highly radiogenic, with ^187^Re/^188^Os ratios ranging from ∼1.4 × 10^4^ to 2.7 × 10^5^ [38,95]. The elemental and isotopic variations in py1, py2 and py3 are consistent with their contrasting morphology (Fig. 7a) and distinct sulfur isotope values (insets in Figs 7d–f). Given these substantial differences in Re–Os isotopic characteristics, careful sample separation is essential for obtaining geologically meaningful ages [38].

### Assumptions in isochron dating and mean square weighted deviation

An important but often overlooked assumption in age calculations—whether using the weighted mean or isochron approach—is that the samples constitute a single statistic population [96]. If one or more samples differ significantly from others—e.g. in their uncertainties or elemental/isotopic compositions—it is essential to assess whether they belong to a single population. The Xinqiao study discussed above [38,93,94] is a classical example on this topic.

Anchoring isochrons to plausible initial ^187^Os/^188^Os ratios may be an option when independent constraints on Os_i_ are available, but the impact of Os_i_ on the isochron age must be rigorously evaluated.

Once a date is obtained, its quality is typically evaluated using the probability of goodness of fit (*p*) or mean squared weighted deviation (MSWD), both of which are a functions of the residual sum of squares (RSS) divided by the degree of freedom, and are inversely correlated (i.e. larger MSWD values correspond to smaller *p*) [97,98].

The distribution of MWSD is governed by the degree of freedom [97]. For large datasets (i.e. *N* > 20), the MSWD distribution approximates a normal distribution with an expected mean of 1, but it is skewed to the left for datasets with *N* < 20. For a dataset with *N* samples, the acceptable MSWD range at the 95% confidence interval is [1$- \small\sqrt {{{2} / {f}}}$, 1+$\small\sqrt {{{2} / {f}}} $], where *f* is the degree of freedom (i.e. *f* = *N* − 2 for linear regression).

It is important to note that MWSD should not be used as a simplistic test of date quality [99]. Although a high MSWD (e.g. >1) may suggest the presence of geological dispersion, potentially violating the assumption of isochron dating, it can also result from incomplete propagation of analytical uncertainties [100]. Conversely, a low MSWD (e.g. <1) may reflect an overestimation of analytical uncertainties [100]. One of the most common errors arises from neglecting the correlation between uncertainties in the ^187^Re/^188^Os and ^187^Os/^188^Os ratios. Since geochronological studies, except for *in situ* geochronology discussed below, often use small-*N* datasets (e.g. *N* < 20), the common practice of using 1 as a universal threshold is not appropriate.

For the same sampleset, ongoing advancements in analytical techniques will yield more precise measurements; consequently, the isochron will exhibit a lower probability of goodness of fit (*p*), or higher MSWD in equivalent [99]. We emphasize that high-precision datasets will become increasingly common in the future and are likely to exhibit elevated MSWD values, which should not be viewed unfavorably by editors and reviewers [99]. Similarly, larger datasets may become more prevalent in the future (see the novel prospect of *in situ* geochronology, discussed below), for which the accepted MSWD threshold is approaching 1.

## KEY ADVANCES IN THE PAST DECADE

### Molybdenite Re–Os dating with a precision of 0.5‰

Molybdenite is the only ore mineral for Mo, and a common phase in porphyry deposits. Unlike zircon U–Pb or biotite Ar–Ar ages, which require assumptions to link measured dates to ore formation, molybdenite Re–Os dates can be directly linked to ore-forming processes without ambiguity. The enrichment of Re in molybdenite—up to the % level (Fig. 2c)—along with negligible common Os [101], permits high-precision dating using Equation (3) as a single-mineral chronometer.

Previous studies have shown that radiogenic ^187^Os may be decoupled from Re within molybdenite crystals, whereby zones with ^187^Os gain or loss yield artificially high or low model ages, respectively [102–104], rendering them unsuitable for accurate dating. Within-crystal diffusion of Os, which is enhanced in larger crystals (e.g. >500 μm) and samples with older ages, has been proposed as an explanation [102,103,105]. In practice, fine-grained molybdenite crystals are preferred to avoid this decoupling problem. If only large grains are available, it is critical to make sure that a suitable aliquot is analyzed, and ideally to sample the entire crystal. Grinding a portion of a big crystal into fine powers, as suggested by some studies in the literature, does not solve the decoupling issue. Further discussion on the decoupling between Re and Os is given below in the *in situ* Re–Os dating section.

**Figure 8.**
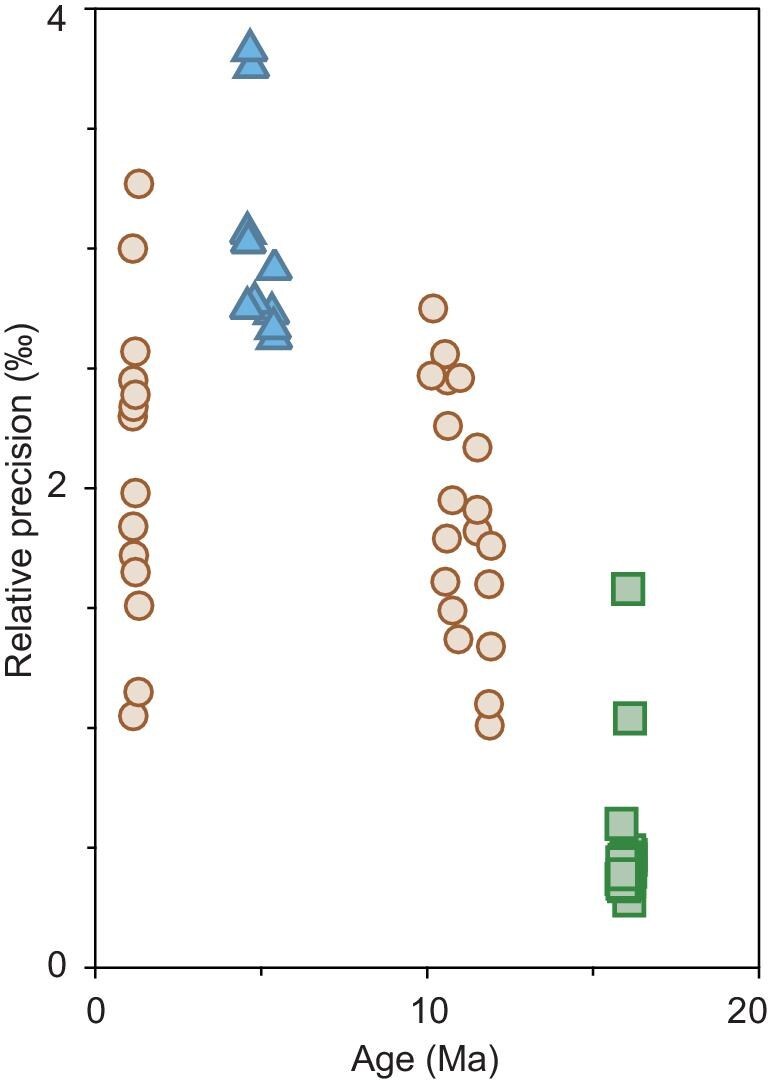
Analytical precision of individual molybdenite Re–Os model ages. Squares represent data from the ∼16 Ma Qulong porphyry deposit, with a reproducibility of ∼0.5‰ using ∼30 mg samples [49]. Circles represent data from the ∼11 Ma Los Pelambres porphyry deposit [108] and the ∼1 Ma OK TEDI porphyry deposit [47] with a reproducibility of ∼2‰ using ∼20 mg samples. Triangles represent data from the ∼5 Ma El Teniente porphyry deposit [48], with a reproducibility of ∼3‰ using 1–3 mg samples.

The analytical precision of molybdenite Re–Os dating is primarily controlled by the amount of Os loaded onto the filament for measurement [106], which depends on sample size, Re content and sample age. An optimized spike-to-sample ratio is also essential [107], necessitating prior determination of the sample's Re concentration. The exact precision achievable is sample-dependent, and modern molybdenite Re–Os geochronology generally can achieve a precision of 3‰ or better than 0.5‰ (Fig. 8) when only analytical uncertainties are considered, e.g. excluding uncertainties from spike calibration and the decay constant [49]. For molybdenites from the ∼11 Ma Los Pelambres porphyry deposit, using a sample size of ∼20 mg, a precision of 1‰–3‰ (Fig. 8) has been achieved [108], equivalent to a temporal resolution of 10–30 kyr. Because the temporal resolution of radiometric dating is maximized for young samples, a similar precision (1‰–3‰; Fig. 8) for the ∼1 Ma OK Tedi porphyry deposit yields a much higher temporal resolution of 1–3 kyr [47]. For the ∼16 Ma Qulong porphyry deposit, using a sample size of ∼30 mg, a precision of 0.3‰–0.5‰ (Fig. 8) was achieved with a temporal resolution of 6 kyr [49], which is five times better than that from previous studies. Using a smaller sample size of 1–3 mg, an order of magnitude lower than previous studies, a precision of ∼3‰ (Fig. 8) has been achieved for the ∼5 Ma El Teniente porphyry deposit [48], equivalent to a temporal resolution of 15 kyr. A reduced sample size, at the expense of precision, avoids the potential of mixing multi-stage grains and significantly expands the applications of this technique.

**Figure 9.**
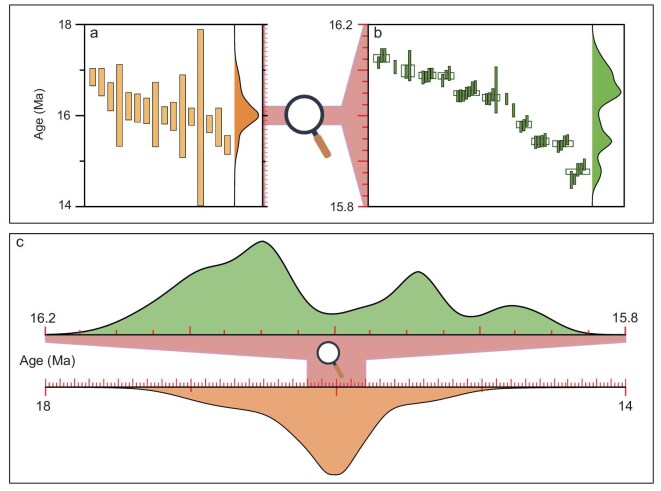
The timescales and rhythms of porphyry copper deposits revealed by molybdenite Re–Os geochronology. (a) Molybdenite Re–Os dates with an analytical precision of 10‰–30‰ (temporal resolution of 160–500 kyr for a 16 Ma system) were used to suggest a continuous mineralization process for the Tibetan Qulong porphyry Cu–Mo deposit between 16.85 and 15.36 Ma, with a duration of ∼1.5 Myr. (b) Molybdenite Re–Os dating with a precision of ∼0.5‰ (temporal resolution of 6 kyr for the 16 Ma system) demonstrates that the ore-forming process is rapid and episodic. (c) The refined duration of 266 kyr is six times shorter than the previous estimate and consists of three intermittent pulses lasting 40–60 kyr [49].

For Re–Os dates with a temporal resolution of a few kyr, we can now begin to decode the cyclic nature of ore-forming processes in giant porphyry systems, as first demonstrated at the Qulong porphyry deposit [49]. This giant porphyry Cu–Mo deposit has been extensively dated by molybdenite Re–Os geochronology, but with a precision of 10‰–30‰ (Fig. 9a), equivalent to a temporal resolution of 160–500 kyr (see references in [109]). The lower-precision dates were used to argue that an extended longevity (i.e. ∼1.5 Myr) is required to form giant deposits. However, through ultra-high precision (0.5‰) Re–Os dating, new dates with a temporal resolution of 6 kyr instead indicate that the giant Qulong deposit was formed within 266 kyr, a 6-fold shorter duration than previously thought (Fig. 9b). The dates further reveal that the ore-forming process is not continuous but comprises three intermittent pulses, each lasting ∼40–60 kyr (Fig. 9c).

A rapid, pulsed nature of the ore-forming process in porphyry copper systems represents a paradigm shift, initially defined by ultra-high precision Re–Os dating [110], which has now been consistently demonstrated across magmatic-hydrothermal systems globally [111–116].

The Qulong example presented in Fig. 9 further highlights that when low-precision geochronological datasets are used, a pulsed process with a hiatus cannot be determined. To investigate the pulsed nature of rapid geological processes, we emphasize that a minimum threshold of analytical precision is required for geochronology [117], and a sufficient number of samples is needed [118].

### Imaging-guided Re–Os dating of organic-rich sediments

Re–Os geochronology is one of the few methods that can directly and robustly date organic-rich sedimentary rocks in the absence of interbedded volcanic ash layers, and is widely applied to provide age information on climate perturbations and biological evolution [29,36,119–125].

Rhenium and Os are highly mobile during weathering [126–128], post-formation alteration and metamorphism [129,130], resulting in open-system behavior for the Re–Os chronometer in organic-rich sediments; therefore, fresh drill core samples are strongly preferred for geochronological studies [131]. To obtain a high-quality isochron, in addition to meeting the prerequisites—i.e. that samples formed simultaneously, share an identical Os_i_ and remained a closed isotopic system—samples also need to display a significant spread in ^187^Re/^188^Os ratios to enable linear regression. This can be achieved by sampling vertically along the drill core, and the height of this vertical profile needs to be sufficiently large; otherwise, the variation in ^187^Re/^188^Os ratios will be limited. However, if the vertical profile is too extended, it may violate the prerequisite assumptions of isochron dating (i.e. identical age and Os_i_).

The sedimentation rates of organic-rich shales are highly variable, with estimates ranging from several centimeters to sub-millimeters per kyr [132–134], corresponding to a thickness of 1–10 m over a 0.1 Ma interval. In this regard, for samples collected from an interval ranging from several tens of centimeters to a few meters, it is generally reasonable to assume a near-synchronous deposition within analytical uncertainties, thereby satisfying the prerequisite of identical formation ages for isochron dating, though compaction should also be considered. However, a broader interval increases the risk of incorporating samples with variable Os_i_ due to changes in Os flux during sedimentation—a concern particularly relevant to organic-rich sediments near stratigraphic boundaries or recording climate perturbations.

**Figure 10.**
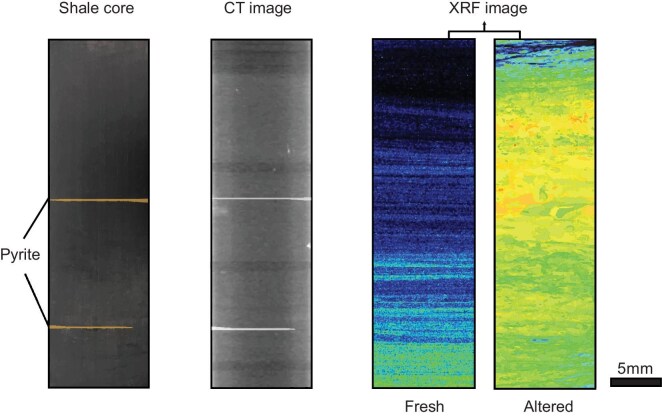
An imaging-guided approach for shale Re–Os geochronology. CT and XRF scanning can be used to aid in selecting the best-preserved shale intervals for Re–Os geochronology akin to cathodoluminescence imaging used in zircon U–Pb geochronology. The shale core and CT image are from the Lantian biota [36]. The Sr distribution maps obtained by XRF are cores from the Qingjiang biota.

To select the best-preserved drill core for Re–Os geochronology, traditional petrographic examination should be conducted to avoid samples with veins, alteration and weathering. Due to the high mobility of Os, low-temperature hydrothermal alteration and/or metamorphism may disturb the Re–Os system in organic-rich sediments, which is often difficult to detect using traditional petrographic approaches, potentially leading to a failed dating attempt [127]. Modern techniques, such as computed tomography (CT) and X-ray fluorescence (XRF), can be used (Fig. 10). A CT scan can produce a 3D distribution of materials with contrasting densities; for example, pyrites and barite, with higher density can be distinguished from less dense materials, such as silicates, pore space and voids [36,42].

Although a CT scan can provide internal information for shales with high spatial resolution, it is unable to determine the chemical distribution of trace elements, which is the major concern for shales that have experienced low-temperature alteration/metamorphism. In contrast, XRF scanning [36] provides a powerful means to assess potential elemental remobilization using elements such as Sr as a proxy (Fig. 10). Furthermore, Re distributions in shales can be obtained using laser ablation inductively coupled plasma mass spectrometry (LA-ICPMS), permitting sampling of the maximum variation in ^187^Re/^188^Os ratios within a given organic-rich sediment interval, which is critical for producing high-quality isochrons. We suggest that an imaging approach using both XRF and potentially LA-ICPMS should be applied for future shale Re–Os geochronology, in a way akin to cathodoluminescence imaging used for zircon U–Pb geochronology.

## OUTLOOK

### *In situ* Re–Os dating

**Figure 11.**
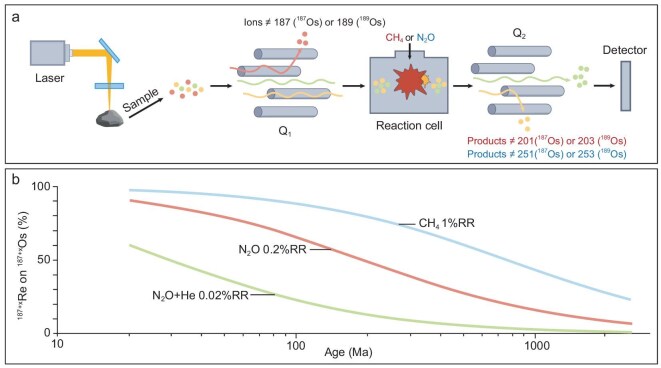
The principles of *in situ* Re–Os dating. (a) A schematic diagram of the mass spectrometer with a reaction cell between two mass analyzers (Q1 and Q2), with samples introduced via a laser ablation system. When CH_4_ is used in the reaction cell, OsCH_2_^+^ is produced and measured at a +14 amu mass shift. When N_2_O is used, OsO_4_^+^ is produced and measured at a +64 amu mass-shift. (b) Rhenium does not react significantly with either gas, but a mathematical interference correction is required to obtain interference-free Os measurements. The expected interference correction of Re on Os is plotted against sample age for a range of typical Re reaction rates in CH_4_, N_2_O and N_2_O + He reaction gas atmospheres. The superscript ‘+x’ represents the mass shifts for the different reaction gases.

A recent innovation in mass spectrometry now enables accurate measurement of isotope ratios free from isobaric interferences, leading to the development of *in situ* beta-decay geochronology [e.g. 135,136]. In this novel approach (Fig. 11), rock blocks or thin sections are ablated by a pulsed laser beam (LA), and the resulting aerosol is atomized and ionized in an inductively coupled plasma (ICP). The ions subsequently travel to a reaction cell, sandwiched between two quadrupole mass analyzers (MS/MS), allowing for accurate, interference-free isotope ratio measurements.

Applied to Re–Os geochronology, the LA-ICP-MS/MS method can effectively separate ^187^Os and ^187^Re by reacting Os with a gas introduced into the reaction cell of the mass spectrometer (Fig. 11a). This method was pioneered by Hogmalm *et al.* [137] and refined by Tamblyn *et al.* [138], demonstrating that Os effectively reacts with CH_4_ gas to form OsCH_2_^+^ ions, while the equivalent reaction of Re to ReCH_2_^+^ is about four orders of magnitude less efficient. However, there is a residual interference that must be accurately corrected by measuring the ^185^ReCH_2_^+^/^185^Re reaction rate in an Os-free material [typically National Institute of Standards and Technology (NIST) glass] and correcting for ^185^Re/^187^Re mass bias using the natural isotopic composition of Re. This ReCH_2_^+^ production rate is typically around 1%–2% [138], which can equate to a significant interference correction on OsCH_2_^+^, especially for young samples with low radiogenic Os ingrowth.

It has been demonstrated that Os also reacts with N_2_O gas to form OsO_4_^+^ [139]. The equivalent production rate of ReO_4_^+^ is nearly an order of magnitude lower compared to that of ReCH_2_^+^ in the CH_4_-based method, leading to reduced interference corrections. Glorie *et al.* [140] subsequently showed that adding He into the reaction cell further suppresses Re-related interferences (Fig. 11b). However, this can come with the trade-off of lower signal sensitivity, and the most suitable analytical recipe is often a compromise between minimizing interference corrections and maintaining sufficient signal sensitivity.

The *in situ* method has proved to be very powerful for rapidly obtaining a large quantity of Re–Os dates on molybdenite, i.e. up to 1000 analyses can be performed within a 24-h analytical session. While uncertainties in individual Re/Os ratios are often much larger compared to conventional negative TIMS (NTIMS) measurements (approximately 2%–5% for Re-rich Precambrian molybdenite; >25% for Cenozoic molybdenite), the sample set is often much larger (typically 40–60 dates per sample), resulting in weighted mean dates with reduced uncertainties. For example, the LA-ICP-MS/MS Re–Os uncertainty of 0.6% (*n* = 37) obtained for the Jinka deposit is only marginally larger than the NTIMS Re–Os uncertainty of 0.5% for the same sample [139]. Similarly, an uncertainty of 0.3% was obtained using LA-ICP-MS/MS [138] for a Re-rich sample (*n* = 71), which is comparable to an uncertainty of 0.2% by NTIMS (*n* = 5).

Despite these advances, within-crystal decoupling between Re and Os [102,103,105] remains a potential concern for the accuracy of *in situ* molybdenite Re–Os dating. X-ray absorption fine structure suggests that Re occurs as Re^4+^ in the Mo site of molybdenite, while radiogenic Os occurs as Os^3+^ and Os^4+^, and does not form secondary Os phases such as OsS_2_ and metallic Os [104]. The smaller ionic radius and lower charge of Os may lead to faster Os diffusion in molybdenite compared to Re [104], as previously proposed [102,103]. Nanoscale secondary ion mass spectrometry (NanoSIMS) imaging was used to argue that ^192^Os is homogeneously distributed in molybdenite and that Re and Os are not decoupled [141]. However, this interpretation is uncertain due to the exceptionally low abundance of common Os (and thus ^192^Os) in molybdenite, and the inability of NanoSIMS to distinguish between ^187^Re and ^187^Os [142]. High-resolution scanning transmission electron microscopy (TEM) analyses demonstrated that both Re and Os are incorporated into molybdenite through isomorphic substitution for Mo, with their distribution controlled by molybdenite precipitation and subsequent metamorphism and deformation [143]. Re–Os isotope mapping for molybdenite using LA-ICPMS/MS from the Merlin deposit shows no age variation across large crystals, at least within the resolution of the mapping approach [138]. However, this is an extremely Re-rich example and more *in situ* mapping is required to evaluate the potential decoupling of Os from Re.

The ability to rapidly and accurately date molybdenite in a petrogenetic context [140,144] opens new opportunities for mineral exploration, especially where fast sample throughput is required. Besides molybdenite, Re-rich pyrite, such as the ones from the Peel River deposit and Nick Prospect, also can be dated using this approach [139]. The application of *in situ* Re–Os geochronology is also being tested on shales [145], though further developments are required.

### Inter-laboratory calibration

The development and refinement of Re–Os geochronology, including chemical and mass spectrometric techniques and optimized workflows over the past several decades, have pushed the precision of molybdenite Re–Os model dates to 0.5‰ (Fig. 7) and shale Re–Os isochron ages to 1%. The most important technical breakthroughs include: (ⅰ) the introduction of NTIMS for improved ionization efficiency of Re and Os [88,89]; (ⅱ) the Carius-tube digestion technique for achieving complete Os isotope equilibration between spike and sample under highly oxidized conditions [82]; (ⅲ) the targeted release of hydrogenous components from shales using CrO_3_–H_2_SO_4_ [1]; and (ⅳ) the preparation of Os gravimetric standards using ammonium hexachloroosmate [(NH_4_)_2_OsCl_6_], along with determination of its stoichiometric composition [9,146].

To facilitate the direct comparison of dates from different laboratories collected over a long period of time, uncertainties must be correctly propagated with careful quantification of random and systematic components. When dates from different laboratories are compared, uncertainties from spike calibration must be considered, especially if spikes are calibrated against different gravimetric standards [146]. For molybdenite Re–Os dating, both spike with normal Os isotope composition and spike using enriched natural isotopes are used. While accurately measuring common Os is important, the currently available double spikes—based on enriched natural isotopes [60,61,146], such as ^186^Os–^190^Os, ^188^Os–^190^Os or ^190^Os–^192^Os—are not ideal compared to double spikes composed of synthetic isotopes, such as the ET2535 spike used in the U–Pb community [147].

High-quality reference materials, including natural samples and synthetic solutions (e.g. age solutions, pre-spiked sample solutions), are also critical for monitoring data quality and enabling inter-laboratory comparison [146,148–152].

An excellent example to monitor and reduce the inter-laboratory uncertainties comes from zircon U–Pb geochronology under the community-driven EarthTime initiative [153,154]. Through distributing a spike calibrated against gravimetric standards that are traceable to the SI unit [147], along with a series of breakthroughs in analytical techniques including chemical abrasion [155], an uncertainty of 0.1% for single-crystal ^206^Pb/^238^U ages was successfully achieved [156], and an ambitious target of 0.01% [157] is now within close reach [158].

Currently, analytical uncertainties for the best molybdenite Re–Os ages are ∼0.5‰, but uncertainties from spike calibration and the decay constant are much larger [13,49]. Without a shared spike and the lack of cross-calibration against other chronometers such as U–Pb, direct comparison of Re–Os dates from different laboratories and with other chronometers must include these uncertainties, which limits applications using the highest temporal resolution available.

We propose that the Re–Os community should be fully integrated into the EarthTime initiative by following the successful model of the U–Pb community. Priority tasks include: (ⅰ) calibrating a shared spike traceable to the SI unit; (ⅱ) developing a data reduction protocol with complete propagation of both random and systematic uncertainties; and (ⅲ) preparing and distributing synthetic solutions with known ages for inter-laboratory evaluation.

### Refining the decay constant

The decay constant fundamentally controls the accuracy of radiometric dates, and its uncertainty must be considered when dates from different radiogenic chronometers are compared (i.e. Re–Os and U–Pb). The currently adopted decay constant of ^187^Re is calculated from iron meteorites [13]. The accuracy of this meteorite-derived decay constant is linked to: (ⅰ) the assumption that the dated meteorites have identical Pb–Pb and Re–Os ages, and (ⅱ) the spike used for Re–Os isotope determination.

Although an uncertainty of 0.31% is reported by Smoliar *et al.* [13], this does not include an additional uncertainty of ∼1.2% from the gravimetric standards used for spike calibration. Unless spikes used for Re–Os dating can be traced back to the study by Smoliar *et al.* [13], an uncertainty of ∼1.2% should be applied. With modern molybdenite Re–Os dating techniques now reaching a precision of <0.5‰, being ∼20 times better than the decay constant uncertainty, reassessing the ^187^Re decay constant, and particularly its uncertainty, should be given the highest priority by the Re–Os community.

The accuracy of the decay constant determined by Smoliar *et al.* [13] has been evaluated using a cross-calibration approach with molybdenites and zircons from a series of Mo-bearing porphyry deposits [159]. In this legacy study, the molybdenite Re–Os dates had a precision of 0.1%–0.3%. Because zircon U–Pb dates were collected before the widespread adoption of chemical abrasion [155], ^207^Pb/^206^Pb dates were used, which have a precision of 0.1%–1.8% [159]. Given the uncertainties at the time, assuming molybdenites and zircons were formed at the same time is valid. With more than a 10-fold increase in the analytical precision for modern molybdenite Re–Os geochronology [47–49], and zircon U–Pb geochronology now achieving 0.01% precision and accuracy [162], as well as an improved understanding on the lifetime of porphyry deposits [49], a refined decay constant with substantially improved precision and accuracy is feasible using modern Re–Os and U–Pb dating techniques.

## Supplementary Material

nwaf300_Supplemental_File

## References

[1] Selby D, Creaser RA. Re-Os geochronology of organic rich sediments: an evaluation of organic matter analysis methods. Chem Geol 2003; 200: 225–40. 10.1016/S0009-2541(03)00199-2

[2] Selby D, Creaser RA, Dewing K et al. Evaluation of bitumen as a ^187^Re-^187^Os geochronometer for hydrocarbon maturation and migration: a test case from the Polaris MVT deposit, Canada. Earth Planet Sci Lett 2005; 235: 1–15. 10.1016/j.epsl.2005.02.018

[3] Selby D, Creaser RA, Fowler MG. Re-Os elemental and isotopic systematics in crude oils. Geochim Cosmochim Acta 2007; 71: 378–86. 10.1016/j.gca.2006.09.005

[4] Stein HJ, Morgan JW, Scherstén A. Re-Os dating of low-level highly radiogenic (LLHR) sulfides: the Harnäs Gold Deposit, Southwest Sweden, records continental-scale tectonic events. Econ Geol 2000; 95: 1657–71.

[5] Tripathy GR, Hannah JL, Stein HJ et al. Radiometric dating of marine-influenced coal using Re-Os geochronology. Earth Planet Sci Lett 2015; 432: 13–23. 10.1016/j.epsl.2015.09.030

[6] Saintilan NJ, Creaser RA, Bookstrom AA. Re-Os systematics and geochemistry of cobaltite (CoAsS) in the Idaho cobalt belt, Belt-Purcell Basin, USA: evidence for middle Mesoproterozoic sediment-hosted Co-Cu sulfide mineralization with Grenvillian and Cretaceous remobilization. Ore Geol Rev 2017; 86: 509–25. 10.1016/j.oregeorev.2017.02.032

[7] Toma J, Creaser RA, Card C et al. Re-Os systematics and chronology of graphite. Geochim Cosmochim Acta 2022; 323: 164–82. 10.1016/j.gca.2022.02.012

[8] Stein HJ, Markey RJ, Morgan JW et al. Highly precise and accurate Re-Os ages for molybdenite from the East Qinling molybdenum belt, Shaanxi Province, China. Econ Geol 1997; 92: 827–35. 10.2113/gsecongeo.92.7-8.827

[9] Selby D, Creaser RA. Re-Os geochronology and systematics in molybdenite from the Endako porphyry molybdenum deposit, British Columbia, Canada. Econ Geol 2001; 96: 197–204. 10.2113/gsecongeo.96.1.197

[10] Mao J-W, Zhang Z-C, Zhang Z-H et al. Re-Os isotopic dating of molybdenites in the Xiaoliugou W (Mo) deposit in the northern Qilian mountains and its geological significance. Geochim Cosmochim Acta 1999; 63: 1815–8.

[11] Tennant S . On two metals, found in the black powder remaining after the solution of platina. Philos Trans R Soc 1804; 94: 411–8.

[12] Ogawa M . Preliminary note on a new element allied to molybdenum. J Coll Sci Imp Univ Tokyo 1908; 16: 1–13.

[13] Smoliar MI, Walker RJ, Morgan JW. Re-Os ages of group IIA, IIIA, IVA, and IVB iron meteorites. Science 1996; 271: 1099–102. 10.1126/science.271.5252.1099

[14] Rudnick RL, Gao S. The composition of the continental crust. In: Holland HD, Turekian KK (eds.). Treatise on Geochemistry. Oxford: Elsevier-Pergamon, 2003, 1–64.

[15] Esser BK, Turekian KK. The osmium isotopic composition of the continental crust. Geochim Cosmochim Acta 1993; 57: 3093–104. 10.1016/0016-7037(93)90296-9

[16] McDonough WF, Sun S-S. The composition of the Earth. Chem Geol 1995; 120: 223–53. 10.1016/0009-2541(94)00140-4

[17] Sun S-S . Chemical composition and origin of the earth's primitive mantle. Geochim Cosmochim Acta 1982; 46: 179–92. 10.1016/0016-7037(82)90245-9

[18] Rudnick RL . Making continental crust. Nature 1995; 378: 571–8. 10.1038/378571a0

[19] Walker RJ . Siderophile elements in tracing planetary formation and evolution. Geochem Persp 2016; 5: 1–145. 10.7185/geochempersp.5.1 PMC6376203 30775285

[20] Suzuki K, Lu Q, Shimizu H et al. Reliable Re-Os age for molybdenite. Geochim Cosmochim Acta 1993; 57: 1625–8. 10.1016/0016-7037(93)90021-N

[21] Du A, Yin N, Sun Y et al. Direct dating of molybdenites using the Re-Os geochronometer. Chi Sci Bull 1993; 38: 1319.

[22] Morelli RM, Creaser RA, Selby D et al. Re-Os sulfide geochronology of the Red Dog sediment-hosted Zn-Pb-Ag deposit, Brooks Range, Alaska. Econ Geol 2004; 99: 1569–76. 10.2113/gsecongeo.99.7.1569

[23] Morelli RM, Creaser R, Selby D et al. Rhenium-osmium geochronology of arsenopyrite in Meguma group gold deposits, Meguma terrane, Nova Scotia, Canada: evidence for multiple gold-mineralizing events. Econ Geol 2005; 100: 1229–42. 10.2113/gsecongeo.100.6.1229

[24] Saintilan NJ, Ikenne M, Bernasconi SM et al. The world's highest-grade cobalt mineralization at Bou Azzer associated with Gondwana supercontinent breakup, Serpentinite and Kellwasser hydrocarbon source rocks. Am J Sci 2023; 323: 12. 10.2475/001c.91400

[25] Saintilan NJ, Selby D, Creaser RA et al. Sulphide Re-Os geochronology links orogenesis, salt and Cu-Co ores in the Central African Copperbelt. Sci Rep 2018; 8: 14946. 10.1038/s41598-018-33399-7 30297732 PMC6175924

[26] Hnatyshin D, Creaser RA, Wilkinson JJ et al. Re-Os dating of pyrite confirms an early diagenetic onset and extended duration of mineralization in the Irish Zn-Pb ore field. Geology 2015; 43: 143–6. 10.1130/G36296.1

[27] Creaser RA, Sannigrahi P, Chacko T et al. Further evaluation of the Re-Os geochronometer in organic-rich sedimentary rocks: a test of hydrocarbon maturation effects in the Exshaw Formation, Western Canada Sedimentary Basin. Geochim Cosmochim Acta 2002; 66: 3441–52. 10.1016/S0016-7037(02)00939-0

[28] Kendall BS, Creaser RA, Ross GM et al. Constraints on the timing of Marinoan “Snowball Earth” glaciation by ^187^Re-^187^Os dating of a Neoproterozoic, post-glacial black shale in Western Canada. Earth Planet Sci Lett 2004; 222: 729–40. 10.1016/j.epsl.2004.04.004

[29] Hannah JL, Bekker A, Stein HJ et al. Primitive Os and 2316 Ma age for marine shale: implications for Paleoproterozoic glacial events and the rise of atmospheric oxygen. Earth Planet Sci Lett 2004; 225: 43–52. 10.1016/j.epsl.2004.06.013

[30] Rooney AD, Macdonald FA, Strauss JV et al. Re-Os geochronology and coupled Os-Sr isotope constraints on the Sturtian snowball Earth. Proc Natl Acad Sci USA 2014; 111: 51–6. 10.1073/pnas.1317266110 24344274 PMC3890860

[31] Selby D, Creaser RA. Direct radiometric dating of hydrocarbon deposits using rhenium-osmium isotopes. Science 2005; 308: 1293–5. 10.1126/science.1111081 15919988

[32] Finlay AJ, Selby D, Osborne MJ et al. Fault-charged mantle-fluid contamination of United Kingdom North Sea oils: insights from Re-Os isotopes. Geology 2010; 38: 979–82. 10.1130/G31201.1

[33] Tessalina SG, Yudovskaya MA, Chaplygin IV et al. Sources of unique rhenium enrichment in fumaroles and sulphides at Kudryavy volcano. Geochim Cosmochim Acta 2008; 72: 889–909. 10.1016/j.gca.2007.11.015

[34] Gao S, Rudnick RL, Carlson RW et al. Re-Os evidence for replacement of ancient mantle lithosphere beneath the North China craton. Earth Planet Sci Lett 2002; 198: 307–22. 10.1016/S0012-821X(02)00489-2

[35] Griffin WL, Spetsius ZV, Pearson NJ et al. *In situ* Re-Os analysis of sulfide inclusions in kimberlitic olivine: new constraints on depletion events in the Siberian lithospheric mantle. Geochem Geophys Geosyst 2002; 3: 1–25.

[36] Yang C, Li Y, Selby D et al. Implications for Ediacaran biological evolution from the ca. 602 Ma Lantian biota in China. Geology 2022; 50: 562–6. 10.1130/G49734.1

[37] Sproson AD, Pogge von Strandmann PAE, Selby D et al. Osmium and lithium isotope evidence for weathering feedbacks linked to orbitally paced organic carbon burial and Silurian glaciations. Earth Planet Sci Lett 2022; 577: 117260. 10.1016/j.epsl.2021.117260

[38] Li Y, Selby D, Li X-H et al. Multisourced metals enriched by magmatic-hydrothermal fluids in stratabound deposits of the Middle-Lower Yangtze River metallogenic belt, China. Geology 2018; 46: 391–4. 10.1130/G39995.1

[39] Morelli R, Creaser RA, Seltmann R et al. Age and source constraints for the giant Muruntau gold deposit, Uzbekistan, from coupled Re-Os-He isotopes in arsenopyrite. Geology 2007; 35: 795–8. 10.1130/G23521A.1

[40] Finlay AJ, Selby D, Osborne MJ. Petroleum source rock identification of United Kingdom Atlantic Margin oil fields and the Western Canadian Oil Sands using platinum, palladium, osmium and rhenium: implications for global petroleum systems. Earth Planet Sci Lett 2012; 313–314: 95–104. 10.1016/j.epsl.2011.11.003

[41] Rooney AD, Hnatyshin D, Toma J et al. Application of the ^187^Re-^187^Os geochronometer to crustal materials: systematics, methodology, data reporting, and interpretation. Geol Soc Am Bull 2024; 136: 4091–129. 10.1130/B37294.1

[42] Stein H, Hannah J. Rhenium–osmium geochronology: sulfides, shales, oils, and mantle. In: Rink WJ, Thompson J (eds.). Encyclopedia of Scientific Dating Methods. Dordrecht: Springer, 2014, 1–25.

[43] Shirey SB, Walker RJ. The Re-Os isotope system in cosmochemistry and high-temperature geochemistry. Annu Rev Earth Planet Sci 1998; 26: 423–500. 10.1146/annurev.earth.26.1.423

[44] Yin L, Zhao P, Liu J et al. Re-Os isotope system in organic-rich samples for dating and tracing: methodology, principle, and application. Earth-Sci Rev 2023; 238: 104317. 10.1016/j.earscirev.2023.104317

[45] Carlson RW, Shirey SB, Schönbächler M. Applications of PGE radioisotope systems in geo- and cosmochemistry. Elements 2008; 4: 239–45. 10.2113/GSELEMENTS.4.4.239

[46] Zimmerman A, Stein HJ, Morgan JW et al. Re–Os geochronology of the El Salvador porphyry Cu–Mo deposit, Chile: tracking analytical improvements in accuracy and precision over the past decade. Geochim Cosmochim Acta 2014; 131: 13–32. 10.1016/j.gca.2014.01.016

[47] Pollard PJ, Jongens R, Stein H et al. Rapid formation of porphyry and skarn copper-gold mineralization in a postsubduction environment: Re-Os and U-Pb geochronology of the Ok Tedi Mine, Papua New Guinea. Econ Geol 2021; 116: 533–58. 10.5382/econgeo.4799

[48] Spencer ET, Wilkinson JJ, Creaser RA et al. The distribution and timing of molybdenite mineralization at the El Teniente Cu-Mo porphyry deposit, Chile. Econ Geol 2015; 110: 387–421. 10.2113/econgeo.110.2.387

[49] Li Y, Selby D, Condon D et al. Cyclic magmatic-hydrothermal evolution in porphyry systems: high-precision U-Pb and Re-Os geochronology constraints on the Tibetan Qulong porphyry Cu-Mo deposit. Econ Geol 2017; 112: 1419–40. 10.5382/econgeo.2017.4515

[50] Liu Y-L, Yang G, Chen J-F et al. Re-Os dating of pyrite from giant Bayan Obo REE-Nb-Fe deposit. Chin Sci Bull 2004; 49: 2627–31. 10.1360/04wd0185

[51] Yu G, Yang G, Chen J-F et al. Re-Os dating of gold-bearing arsenopyrite of the Maoling gold deposit, Liaoning Province, Northeast China and its geological significance. Chin Sci Bull 2005; 50: 1509–14. 10.1360/04wd0229

[52] Arne DC, Bierlin FP, Morgan JW et al. Re-Os dating of sulfides associated with gold mineralization in central Victoria, Australia. Econ Geol 2001; 96: 1455–9. 10.2113/gsecongeo.96.6.1455

[53] Selby D, Kelley KD, Hitzman MW et al. Re-Os sulfide (bornite, chalcopyrite, and pyrite) systematics of the carbonate-hosted copper deposits at Ruby Creek, Southern Brooks Range, Alaska. Econ Geol 2009; 104: 437–44. 10.2113/gsecongeo.104.3.437

[54] Saintilan NJ, Creaser RA. A Permian rhenium–osmium radiometric age for bornite at the Rock Creek deposit, Spar Lake copper–silver district (Montana, USA)—a link to the Sonoma Orogeny and the copper–silver–vanadium Midcontinent Belt? Can J Earth Sci 2024; 61: 145–57. 10.1139/cjes-2023-0024

[55] Zhu Z, Sun Y. Direct Re-Os dating of chalcopyrite from the Lala IOCG deposit in the Kangdian Copper Belt, China. Econ Geol 2013; 108: 871–82.

[56] Li WJ, Gao BY, Zhang LC et al. LLHR-type chalcopyrite Re-Os geochronology and geochemistry of the Xiaotongchang Cu deposit in south Ailaoshan and its geological significances. J Geochem Explor 2019; 198: 123–31. 10.1016/j.gexplo.2018.11.011

[57] Deng X-H, Wang J-B, Pirajno F et al. Re–Os dating of chalcopyrite from selected mineral deposits in the Kalatag district in the eastern Tianshan Orogen, China. Ore Geol Rev 2016; 77: 72–81. 10.1016/j.oregeorev.2016.01.014

[58] Hnatyshin D, Creaser RA, Meffre S et al. Understanding the microscale spatial distribution and mineralogical residency of Re in pyrite: examples from carbonate-hosted Zn-Pb ores and implications for pyrite Re-Os geochronology. Chem Geol 2020; 533: 119427. 10.1016/j.chemgeo.2019.119427

[59] Anbar AD, Buick R, Gordon GW et al. Technical comment on “Reexamination of 2.5-Ga ‘whiff’ of oxygen interval points to anoxic ocean before GOE”. Sci Adv 2023; 9: eabq3736. 10.1126/sciadv.abq3736 37027472 PMC10081836

[60] Markey R, Hannah JL, Morgan JW et al. A double spike for osmium analysis of highly radiogenic samples. Chem Geol 2003; 200: 395–406. 10.1016/S0009-2541(03)00197-9

[61] Qu W, Du A, Zhao D. Determination of ^187^Os in molybdenite by ICP-MS with neutron-induced ^186^Os and ^188^Os spikes. Talanta 2001; 55: 815–20. 10.1016/S0039-9140(01)00506-9 18968429

[62] Li Y, Zhang S, Hobbs R et al. Monte Carlo sampling for error propagation in linear regression and applications in isochron geochronology. Sci Bull 2019; 64: 189–97. 10.1016/j.scib.2018.12.019 36659617

[63] Li Y, Vermeesch P. Inverse isochron regression for Re–Os, K–Ca and other chronometers. Geochronology 2021; 3: 415–20. 10.5194/gchron-3-415-2021

[64] Day JMD, Kelly S, Troll VR et al. Deep crustal assimilation during the 2021 Fagradalsfjall Fires, Iceland. Nature 2024; 632: 564–9. 10.1038/s41586-024-07750-0 39085608

[65] Oxburgh R . Residence time of osmium in the oceans. Geochem Geophys Geosyst 2001; 2: 1018. 10.1029/2000GC000104

[66] Levasseur S, Birck JL, Allègre CJ. The osmium riverine flux and the oceanic mass balance of osmium. Earth Planet Sci Lett 1999; 174: 7–23. 10.1016/S0012-821X(99)00259-9

[67] Rooney AD, Selby D, Lloyd JM et al. Tracking millennial-scale Holocene glacial advance and retreat using osmium isotopes: insights from the Greenland ice sheet. Quat Sci Rev 2016; 138: 49–61. 10.1016/j.quascirev.2016.02.021

[68] Ownsworth E, Selby D, Lloyd J et al. Tracking sediment delivery to central Baffin Bay during the past 40 kyrs: insights from a multiproxy approach and new age model. Quat Sci Rev 2023; 308: 108082. 10.1016/j.quascirev.2023.108082

[69] Du Vivier ADC, Selby D, Condon DJ et al. Pacific ^187^Os/^188^Os isotope chemistry and U–Pb geochronology: synchroneity of global Os isotope change across OAE 2. Earth Planet Sci Lett 2015; 428: 204–16. 10.1016/j.epsl.2015.07.020

[70] Turgeon SC, Creaser RA. Cretaceous oceanic anoxic event 2 triggered by a massive magmatic episode. Nature 2008; 454: 323–6. 10.1038/nature07076 18633415

[71] Tejada MLG, Suzuki K, Kuroda J et al. Ontong Java Plateau eruption as a trigger for the early Aptian oceanic anoxic event. Geology 2009; 37: 855–8. 10.1130/G25763A.1

[72] Cohen AS, Coe AL, Harding SM et al. Osmium isotope evidence for the regulation of atmospheric CO_2_ by continental weathering. Geology 2004; 32: 157–60. 10.1130/G20158.1

[73] Scott E . Weathering Ediacaran evolution. Nat Rev Earth Environ 2022; 3: 160. 10.1038/s43017-022-00274-z

[74] Zaiss J, Ravizza G, Goderis S et al. A complete Os excursion across a terrestrial Cretaceous–Paleogene boundary at the West Bijou Site, Colorado, with evidence for recent open system behavior. Chem Geol 2014; 385: 7–16. 10.1016/j.chemgeo.2014.07.010

[75] Luck J-M, Allègre CJ. ^187^Re–^187^Os systematics in meteorites and cosmochemical consequences. Nature 1983; 302: 130–2. 10.1038/302130a0

[76] Palme H . Platinum-group elements in cosmochemistry. Elements 2008; 4: 233–8. 10.2113/GSELEMENTS.4.4.233

[77] Dalai TK, Ravizza GE, Peucker-Ehrenbrink B. The late Eocene ^187^Os/^188^Os excursion: chemostratigraphy, cosmic dust flux and the early Oligocene glaciation. Earth Planet Sci Lett 2006; 241: 477–92. 10.1016/j.epsl.2005.11.035

[78] Koeberl C, Shirey SB. Re–Os isotope systematics as a diagnostic tool for the study of impact craters and distal ejecta. Palaeogeogr Palaeoclimatol Palaeoecol 1997; 132: 25–46. 10.1016/S0031-0182(97)00045-X

[79] Paquay FS, Ravizza GE, Dalai TK et al. Determining chondritic impactor size from the marine osmium isotope record. Science 2008; 320: 214–8. 10.1126/science.1152860 18403707

[80] Reisberg L, Meisel T. The Re-Os isotopic system: a review of analytical techniques. Geostand Newsl 2002; 26: 249–67. 10.1111/j.1751-908X.2002.tb00633.x

[81] Ishikawa A, Senda R, Suzuki K et al. Re-evaluating digestion methods for highly siderophile element and ^187^Os isotope analysis: evidence from geological reference materials. Chem Geol 2014; 384: 27–46. 10.1016/j.chemgeo.2014.06.013

[82] Shirey SB, Walker RJ. Carius tube digestion for low-blank rhenium-osmium analysis. Anal Chem 1995; 67: 2136–41. 10.1021/ac00109a036

[83] Pearson DG, Woodland SJ. Solvent extraction/anion exchange separation and determination of PGEs (Os, Ir, Pt, Pd, Ru) and Re–Os isotopes in geological samples by isotope dilution ICP-MS. Chem Geol 2000; 165: 87–107. 10.1016/S0009-2541(99)00161-8

[84] Cohen AS, Waters FG. Separation of osmium from geological materials by solvent extraction for analysis by thermal ionisation mass spectrometry. Anal Chim Acta 1996; 332: 269–75. 10.1016/0003-2670(96)00226-7

[85] Li C, Qu W-J, Du A-D et al. Comprehensive study on extraction of rhenium with acetone in Re-Os isotopic dating (in Chinese with English abstract). Rock Miner Anal 2009; 28: 233–8.

[86] Wang L-B, Qu W-J, Li C et al. Method study on the separation and enrichment of rhenium measured by negative thermal ionization mass spectrometry (in Chinese with English abstract). Rock Miner Anal 2013; 32: 402–8.

[87] Hassler DR, Peucker-Ehrenbrink B, Ravizza GE. Rapid determination of Os isotopic composition by sparging OsO_4_ into a magnetic-sector ICP-MS. Chem Geol 2000; 166: 1–14. 10.1016/S0009-2541(99)00180-1

[88] Creaser RA, Papanastassiou DA, Wasserburg GJ. Negative thermal ion mass-spectrometry of osmium, rhenium, and iridium. Geochim Cosmochim Acta 1991; 55: 397–401. 10.1016/0016-7037(91)90427-7

[89] Völkening J, Walczyk T, Heumann KG. Osmium isotope ratio determinations by negative thermal ionization mass spectrometry. Int J Mass Spectrom Ion Processes 1991; 105: 147–59. 10.1016/0168-1176(91)80077-Z

[90] Wendt I . Isochron or mixing line. Chem Geol 1993; 104: 301–5. 10.1016/0009-2541(93)90159-G

[91] Davidson J, Charlier B, Hora JM. Mineral isochrons and isotopic fingerprinting: pitfalls and promises. Geology 2005; 33: 29–32. 10.1130/G21063.1

[92] Zheng YF . Influences of the nature of the initial Rb-Sr system on isochron validity. Chem Geol 1989; 80: 1–16.

[93] Li Y, Li QL, Yang JH. Tracing water-rock interaction in carbonate replacement deposits: a SIMS pyrite S-Pb isotope perspective from the Chinese Xinqiao system. Ore Geol Rev 2019; 107: 248–57. 10.1016/j.oregeorev.2019.02.022

[94] Li Y, Li JW, Li XH et al. An early cretaceous carbonate replacement origin for the Xinqiao stratabound massive sulfide deposit, Middle-Lower Yangtze Metallogenic Belt, China. Ore Geol Rev 2017; 80: 985–1003. 10.1016/j.oregeorev.2016.08.017

[95] Guo WM, Lu JJ, Jiang SY et al. Re-Os isotope dating of pyrite from the footwall mineralization zone of the Xinqiao deposit, Tongling, Anhui Province: geochronological evidence for submarine exhalative sedimentation. Chin Sci Bull 2011; 56: 3860–5. 10.1007/s11434-011-4770-y

[96] York D, Evensen NM, Martinez ML et al. Unified equations for the slope, intercept, and standard errors of the best straight line. Am J Phys 2004; 72: 367–75. 10.1119/1.1632486

[97] Wendt I, Carl C. The statistical distribution of the mean squared weighted deviation. Chem Geol 1991; 86: 275–85.

[98] Kalsbeek F . The statistical distribution of the mean squared weighted deviation—Comment: Isochrons, errorchrons, and the use of MSWD-values. Chem Geol 1992; 94: 241–2. 10.1016/S0009-2541(10)80008-7

[99] Vermeesch P . High MSWDs are not the problem, low ones are. Natl Sci Rev 2025; 12: nwaf036. 10.1093/nsr/nwaf036 40809878 PMC12342530

[100] Ludwig KR . User's Manual for Isoplot 3.00: a Geochronological Toolkit for Microsoft Excel. https://manualzz.com/doc/7052840/berkeley-geochronology-center-isoplot-geochronological-to...#related (7 August 2025, date last accessed).

[101] Stein HJ, Markey RJ, Morgan JW et al. The remarkable Re-Os chronometer in molybdenite: how and why it works. Terra Nova 2001; 13: 479–86. 10.1046/j.1365-3121.2001.00395.x

[102] Selby D, Creaser RA. Macroscale NTIMS and microscale LA-MC-ICP-MS Re-Os isotopic analysis of molybdenite: testing spatial restrictions for reliable Re-Os age determinations, and implications for the decoupling of Re and Os within molybdenite. Geochim Cosmochim Acta 2004; 68: 3897–908. 10.1016/j.gca.2004.03.022

[103] Stein H, Scherstén A, Hannah J et al. Subgrain-scale decoupling of Re and ^187^Os and assessment of laser ablation ICP-MS spot dating in molybdenite. Geochim Cosmochim Acta 2003; 67: 3673–86. 10.1016/S0016-7037(03)00269-2

[104] Takahashi Y, Uruga T, Suzuki K et al. An atomic level study of rhenium and radiogenic osmium in molybdenite. Geochim Cosmochim Acta 2007; 71: 5180–90. 10.1016/j.gca.2007.08.007

[105] Selby D, Creaser RA, Feely M. Accurate and precise Re-Os molybdenite dates from the Galway Granite, Ireland. Critical comment on “Disturbance of the Re-Os chronometer of molybdenites from the late-Caledonian Galway Granite, Ireland, by hydrothermal fluid circulation” by Suzuki *et al.*, Geochemical Journal, 35, 29-35, 2001. Geochem J 2004; 38: 291–4.

[106] Nanne JAM, Millet MA, Burton KW et al. High precision osmium stable isotope measurements by double spike MC-ICP-MS and N-TIMS. J Anal At Spectrom 2017; 32: 749–65. 10.1039/C6JA00406G

[107] John SG . Optimizing sample and spike concentrations for isotopic analysis by double-spike ICPMS. J Anal At Spectrom 2012; 27: 2123–31. 10.1039/c2ja30215b

[108] Stein HJ . Dating and tracing the history of ore formation. In: Turekian HDHK (ed.). Treatise on Geochemistry, 2nd edn. Oxford: Elsevier, 2014, 87–118.

[109] Li Y, Selby D, Feely M et al. Fluid inclusion characteristics and molybdenite Re-Os geochronology of the Qulong porphyry copper-molybdenum deposit, Tibet. Miner Depos 2017; 52: 137–58. 10.1007/s00126-016-0654-z

[110] Chiaradia M, Mathur R, Vennemann T et al. Applications of radiogenic and transition metal isotopes to the study of metallic mineral deposits. In: Anbar A, Weis D (eds). Treatise on Geochemistry, 3rd edn. Oxford: Elsevier, 2025, 949–1018.

[111] Li Y, Allen MB, Li XH. Millennial pulses of ore formation and an extra-high Tibetan Plateau. Geology 2022; 50: 665–9. 10.1130/G49911.1

[112] Li Y, Zhang RQ, He S et al. Pulsed exsolution of magmatic ore-forming fluids in tin-tungsten systems: a SIMS cassiterite oxygen isotope record. Miner Depos 2022; 57: 343–52. 10.1007/s00126-022-01093-4

[113] Tan W, Reddy SM, Fougerouse D et al. Superimposed microstructures of pyrite in auriferous quartz veins as fingerprints of episodic fluid infiltration in the Wulong Lode gold deposit, NE China. Miner Depos 2022; 57: 685–700. 10.1007/s00126-022-01104-4

[114] Jiang XJ, Ma Y, Kang YM et al. Determining multiple fluid pulse and evolution using zoned garnet in Mengya'a skarn Pb-Zn-polymetallic deposit, Tibet. Ore Geol Rev 2023; 163: 105795. 10.1016/j.oregeorev.2023.105795

[115] Steadman JA, Large RR, Olin PH et al. Pyrite trace element behavior in magmatic-hydrothermal environments: an LA-ICPMS imaging study. Ore Geol Rev 2021; 128: 103878. 10.1016/j.oregeorev.2020.103878

[116] Del Real I, Reich M, Simon AC et al. Formation of giant iron oxide-copper-gold deposits by superimposed, episodic hydrothermal pulses. Commun Earth Environ 2021; 2: 192. 10.1038/s43247-021-00265-w PMC10368639 37491481

[117] Schoene B, Eddy MP, Keller CB et al. An evaluation of Deccan Traps eruption rates using geochronologic data. Geochronology 2021; 3: 181–98. 10.5194/gchron-3-181-2021

[118] Glazner AF, Sadler PM. Estimating the duration of geologic intervals from a small number of age determinations: a challenge common to petrology and paleobiology. Geochem Geophys Geosyst 2016; 17: 4892–8. 10.1002/2016GC006542

[119] Kendall BS, Creaser RA, Ross GM et al. Constraints on the timing of Marinoan “Snowball Earth” glaciation by ^187^Re–^187^Os dating of a Neoproterozoic, post-glacial black shale in Western Canada. Earth Planet Sci Lett 2004; 222: 729–40. 10.1016/j.epsl.2004.04.004

[120] Kendall B, Creaser RA, Selby D. Re-Os geochronology of postglacial black shales in Australia: constraints on the timing of “Sturtian” glaciation. Geology 2006; 34: 729–32. 10.1130/G22775.1

[121] Strauss JV, Rooney AD, Macdonald FA et al. 740 Ma vase-shaped microfossils from Yukon, Canada: implications for Neoproterozoic chronology and biostratigraphy. Geology 2014; 42: 659–62. 10.1130/G35736.1

[122] Rooney AD, Strauss JV, Brandon AD et al. A Cryogenian chronology: two long-lasting synchronous Neoproterozoic glaciations. Geology 2015; 43: 459–62. 10.1130/G36511.1

[123] Xu WM, Ruhl M, Jenkyns HC et al. Carbon sequestration in an expanded lake system during the Toarcian oceanic anoxic event. Nat Geosci 2017; 10: 129–34. 10.1038/ngeo2871

[124] Finlay AJ, Selby D, Gröcke DR. Tracking the Hirnantian glaciation using Os isotopes. Earth Planet Sci Lett 2010; 293: 339–48. 10.1016/j.epsl.2010.02.049

[125] Selby D, Creaser RA. Direct radiometric dating of the Devonian-Mississippian time-scale boundary using the Re-Os black shale geochronometer. Geology 2005; 33: 545–8. 10.1130/G21324.1

[126] Peucker-Ehrenbrink B, Hannigan RE. Effects of black shale weathering on the mobility of rhenium and platinum group elements. Geology 2000; 28: 475–8. 10.1130/0091-7613(2000)28<475:EOBSWO>2.0.CO;2

[127] Georgiev S, Stein HJ, Hannah JL et al. Chemical signals for oxidative weathering predict Re–Os isochroneity in black shales, East Greenland. Chem Geol 2012; 324–325: 108–21. 10.1016/j.chemgeo.2012.01.003

[128] Jaffe LA, Peucker-Ehrenbrink B, Petsch ST. Mobility of rhenium, platinum group elements and organic carbon during black shale weathering. Earth Planet Sci Lett 2002; 198: 339–53. 10.1016/S0012-821X(02)00526-5

[129] Tripathy GR, Singh SK, Bhu H. Re–Os isotopes and major and trace element geochemistry of carbonaceous shales, Aravalli Supergroup, India: impact of post-depositional processes Chem Geol 2013; 354: 93–106. 10.1016/j.chemgeo.2013.06.014

[130] Pierson-Wickmann A-C, Reisberg L, France-Lanord C. Behavior of Re and Os during low-temperature alteration: results from Himalayan soils and altered black shales. Geochim Cosmochim Acta 2002; 66: 1539–48. 10.1016/S0016-7037(01)00865-1

[131] Kendall B, Creaser RA, Selby D. ^187^Re-^187^Os geochronology of Precambrian organic-rich sedimentary rocks. Geol Soc Spec Publ 2009; 326: 85–107. 10.1144/SP326.5

[132] Piper DZ, Calvert SE. A marine biogeochemical perspective on black shale deposition. Earth-Sci Rev 2009; 95: 63–96. 10.1016/j.earscirev.2009.03.001

[133] Sadler PM . Sediment accumulation rates and the completeness of stratigraphic sections. J Geol 1981; 89: 569–84. 10.1086/628623

[134] Tyson RV . Sedimentation rate, dilution, preservation and total organic carbon: some results of a modelling study. Org Geochem 2001; 32: 333–9. 10.1016/S0146-6380(00)00161-3

[135] Zack T, Gilbert SE. Chapter 7–*In situ* beta decay dating by LA-ICP-MS/MS: fundamentals and methodology. In: Shellnutt JG, Denyszyn SW, Suga K (eds.). Methods and Applications of Geochronology. Amsterdam: Elsevier, 2024, 211–41.

[136] Gilbert SE, Glorie S, Zack T. Chapter 8–*In situ* beta decay dating by LA-ICP-MS/MS: applications. In: Shellnutt JG, Denyszyn SW, Suga K (eds.). Methods and Applications of Geochronology. Amsterdam: Elsevier, 2024, 243–95.

[137] Hogmalm KJ, Dahlgren I, Fridolfsson I et al. First in situ Re-Os dating of molybdenite by LA-ICP-MS/MS. Miner Depos 2019; 54: 821–8. 10.1007/s00126-019-00889-1

[138] Tamblyn R, Gilbert S, Glorie S et al. Molybdenite reference materials for *in situ* LA-ICP-MS/MS Re-Os geochronology. Geostand Geoanal Res 2024; 48: 393–410. 10.1111/ggr.12550

[139] Simpson A, Glorie S, Gilbert S et al. LA-ICP-Q-MS/MS Re–Os geochronology: a comparison of N_2_O and CH_4_ reaction gases. Chem Geol 2024; 670: 122384. 10.1016/j.chemgeo.2024.122384

[140] Glorie S, Thompson JM, Gilbert SE et al. *In situ* Re–Os geochronology of Re-rich Palaeogene molybdenite by LA-ICP-MS/MS. J Anal At Spectrom 2025; 40: 1394–402. 10.1039/D5JA00030K

[141] Barra F, Deditius A, Reich M et al. Dissecting the Re-Os molybdenite geochronometer. Sci Rep 2017; 7: 16054. 10.1038/s41598-017-16380-8 29167505 PMC5700062

[142] Zimmerman A, Yang G, Stein HJ et al. A critical review of molybdenite ^187^Re parent-^187^Os daughter intra-crystalline decoupling in light of recent *in situ* micro-scale observations. Geostand Geoanal Res 2022; 46: 761–72. 10.1111/ggr.12448

[143] Tan M, Yang YP, Huang XW et al. Rhenium residency in molybdenite, compressional textures and relationship to polytypism. Geochim Cosmochim Acta 2025; 389: 59–73. 10.1016/j.gca.2024.12.012

[144] Simpson A, Glorie S, Hand M et al. *In situ* apatite and carbonate Lu-Hf and molybdenite Re-Os geochronology for ore deposit research: method validation and example application to Cu-Au mineralisation. Geosci Front 2024; 15: 101867. 10.1016/j.gsf.2024.101867

[145] Thompson JM, Emsbo P, Souders K et al. In-situ dating of black shales with the Re-Os geochronometer using LA-ICP-MS/MS. Goldschmidt 2024 Conference, Chicago, 22 August 2024.

[146] Markey R, Stein HJ, Hannah JL et al. Standardizing Re-Os geochronology: a new molybdenite reference material (Henderson, USA) and the stoichiometry of Os salts. Chem Geol 2007; 244: 74–87. 10.1016/j.chemgeo.2007.06.002

[147] Condon DJ, Schoene B, McLean NM et al. Metrology and traceability of U-Pb isotope dilution geochronology (EARTHTIME Tracer Calibration Part I). Geochim Cosmochim Acta 2015; 164: 464–80. 10.1016/j.gca.2015.05.026

[148] Du AD, Wu SQ, Sun DZ et al. Preparation and certification of Re-Os dating reference materials: molybdenites HLP and JDC. Geostand Geoanal Res 2004; 28: 41–52. 10.1111/j.1751-908X.2004.tb01042.x

[149] Wang MJ, Chu ZY, Meisel TC et al. Determination of Re, Os, Ir, Ru, Pt, Pd mass fractions and ^187^Os/^188^Os ratios of organic-rich geological reference materials. Geostand Geoanal Res 2022; 46: 333–49. 10.1111/ggr.12423

[150] Liu JJ, Selby D. A matrix-matched reference material for validating petroleum Re-Os measurements. Geostand Geoanal Res 2018; 42: 97–113. 10.1111/ggr.12193

[151] Li J, Yin L. Rhenium–osmium isotope measurements in marine shale reference material SBC-1: implications for method validation and quality control. Geostand Geoanal Res 2019; 43: 497–507. 10.1111/ggr.12267

[152] Luguet A, Nowell GM, Pearson DG. ^184^Os/^188^Os and ^186^Os/^188^Os measurements by negative thermal ionisation mass spectrometry (N-TIMS): effects of interfering element and mass fractionation corrections on data accuracy and precision. Chem Geol 2008; 248: 342–62. 10.1016/j.chemgeo.2007.10.013

[153] Bowring SA, Erwin D, Parrish R et al. EARTHTIME: a community-based effort towards high-precision calibration of earth history. Geochim Cosmochim Acta 2005; 69: A316.

[154] Condon D, Schoene B, Bowring S et al. EARTHTIME: isotopic tracers and optimized solutions for high-precision U-Pb ID-TIMS geochronology. Abstract V41E-06, American Geophysical Union Fall Meeting, San Francisco, 10–14 December 2007.

[155] Mattinson JM . Zircon U–Pb chemical abrasion (“CA-TIMS”) method: combined annealing and multi-step partial dissolution analysis for improved precision and accuracy of zircon ages. Chem Geol 2005; 220: 47–66. 10.1016/j.chemgeo.2005.03.011

[156] Schaltegger U, Ovtcharova M, Gaynor SP et al. Long-term repeatability and interlaboratory reproducibility of high-precision ID-TIMS U-Pb geochronology. J Anal At Spectrom 2021; 36: 1466–77. 10.1039/D1JA00116G 34276120 PMC8262554

[157] National Academies of Sciences E, Medicine . A Vision for NSF Earth Sciences 2020–2030: Earth in Time. Washington DC: National Academies Press, 2020.

[158] Szymanowski D, Wotzlaw J-F, Ovtcharova M et al. Interlaboratory reproducibility of ID-TIMS U–Pb geochronology evaluated with a pre-spiked natural zircon solution. EGUsphere [Preprint]. 10.5194/egusphere-2025-1001.

[159] Selby D, Creaser RA, Stein HJ et al. Assessment of the Re decay constant by cross calibration of Re-Os molybdenite and U-Pb zircon chronometers in magmatic ore systems. Geochim Cosmochim Acta 2007; 71: 1999–2013. 10.1016/j.gca.2007.01.008

[160] Schmitz MD, Schoene B. Derivation of isotope ratios, errors, and error correlations for U-Pb geochronology using ^205^Pb-^235^U-(^233^U)-spiked isotope dilution thermal ionization mass spectrometric data. Geochem Geophys Geosyst 2007; 8: Q08006. 10.1029/2006GC001492

[161] Ludwig KR . Calculation of uncertainties of U-Pb isotope data. Earth Planet Sci Lett 1980; 46: 212–20. 10.1016/0012-821X(80)90007-2

[162] Yang C, Bowyer F, Condon D. High-precision CA-ID-TIMS zircon U-Pb geochronology: a review of the Neoproterozoic time scale. Natl Sci Rev 2025; 12: nwaf206. 10.1093/nsr/nwaf206 40809879 PMC12342614

